# Supplementary material for: Fabrication of p-type 2D single-crystalline transistor arrays with Fermi-level-tuned van der Waals semimetal electrodes
Source: Nat Commun. 2023 Aug 7;14:4747. doi: 10.1038/s41467-023-40448-x (PMC10406929; doi:10.1038/s41467-023-40448-x)
Supplement: Supplementary file 1 — Supplementary Information [file 41467_2023_40448_MOESM1_ESM.pdf]

Supplementary information for:

Fabrication of *p*-type 2D single-crystalline transistor arrays with Fermi-level-tuned van der Waals semimetal electrodes

Seunguk Song[1,2,†], Aram Yoon[1,3,†], Sora Jang[1,†,], Jason Lynch[2], Jihoon Yang[1], Juwon Han[1], Myeonggi Choe[1,3], Young Ho Jin[1], Yueli Chen[4], Yeryun Cheon[5], Jinsung Kwak[1,6], Changwook Jeong[1], Hyeonsik Cheong[5], Deep Jariwala[2], Zonghoon Lee[1,3*], and Soon-Yong Kwon[1*]

[1]*Department of Materials Science and Engineering & Graduate School of Semiconductor Materials and Devices Engineering, Ulsan National Institute of Science and Technology (UNIST), Ulsan 44919, Republic of Korea*
[2]*Department of Electrical and Systems Engineering, University of Pennsylvania, Philadelphia, Pennsylvania 19104, United States*
[3]*Center for Multidimensional Carbon Materials (CMCM), Institute for Basic Science (IBS), Ulsan 44919, Republic of Korea*
[4]*Department of Chemistry, University of Pennsylvania, Philadelphia, Pennsylvania 19104, United States*
[5]*Department of Physics, Sogang University, Seoul 04107, Republic of Korea*
[6]*Department of Physics, Changwon National University, Changwon 51140, Republic of Korea.,*

[†]These authors contributed equally: Seunguk Song, Aram Yoon, Sora Jang.
[*]Correspondence should be addressed. Email to: zhlee@unist.ac.kr (Z.L.), sykwon@unist.ac.kr (S.-Y.K.)

- Supplementary Figs. 1-28
- Supplementary Tables 1-4
- Supplementary References 1-81

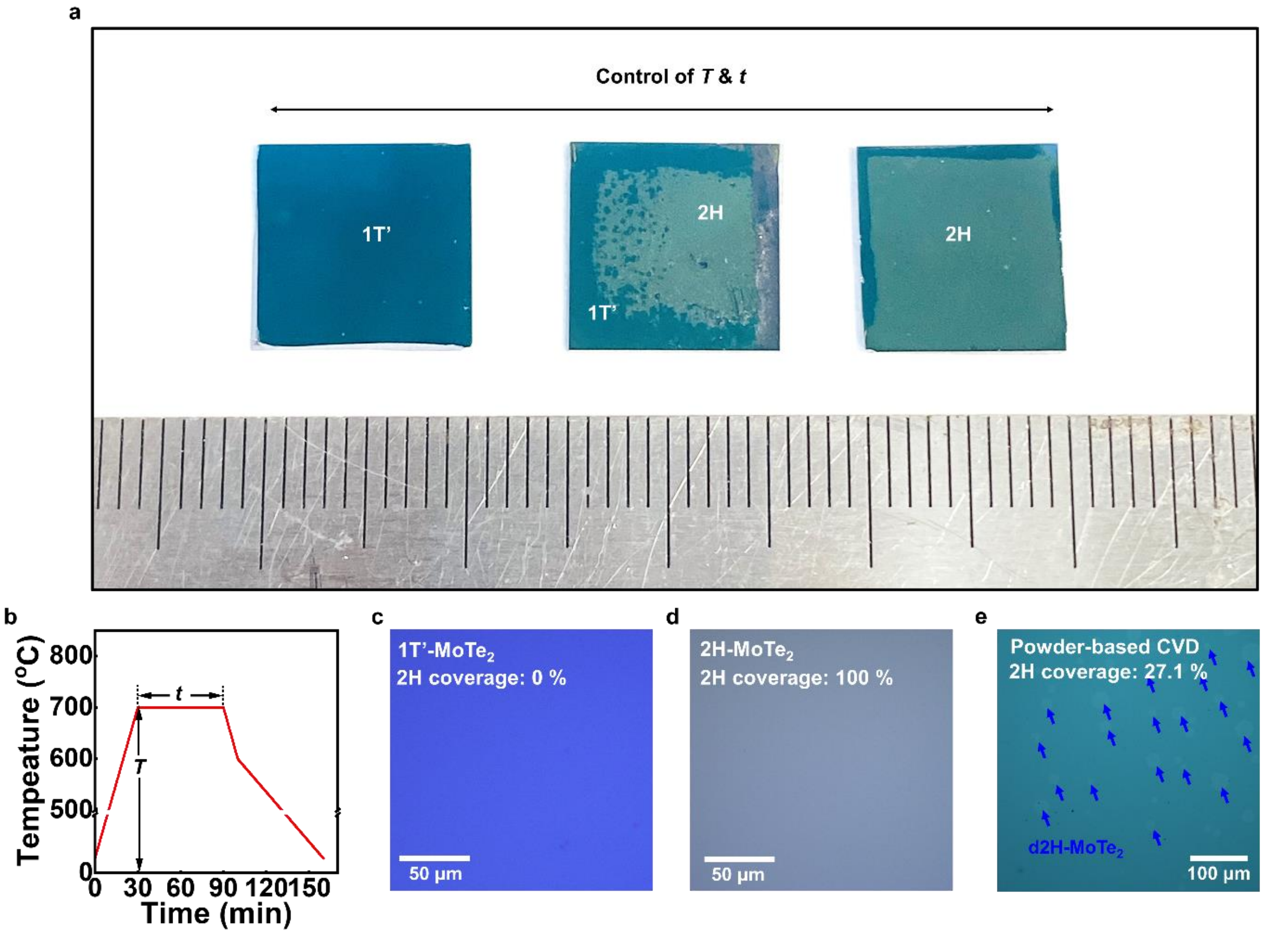

Supplementary Fig. 1. Centimeter-scale growth of $MoTe_2$ thin film on a SiO_2/Si substrate. (**a**) Optical images of the as-grown $MoTe_2$ thin films (H = 7.6 nm), where the 1T' and 2H phases were characteristically different with regard to the color contrast. The dominant phase of the thin films was controlled in-situ by the T and t. The temperature profile of the synthesis apparatus is depicted in (**b**). Because the $MoTe_2$ was obtained primarily from Te vapor emitted by the stacked Ni_xTe_y precursor, the phase transformation began at the center of the sample. (**c, d**) OM images of the $MoTe_2$ thin films with fully covered (**c**) 1T' and (**d**) 2H phase. (**e**) OM image of the thin film synthesized using the conventionally used Te powder precursor for the horizontal CVD at T = 700 °C instead of our Te-confined growth mode. By using Te powder instead of the Ni_xTe_y stack, the defective 2H $MoTe_2$ (d2H) was obtained (see details on the d2H-phase in Supplementary Figs. 2m-o).

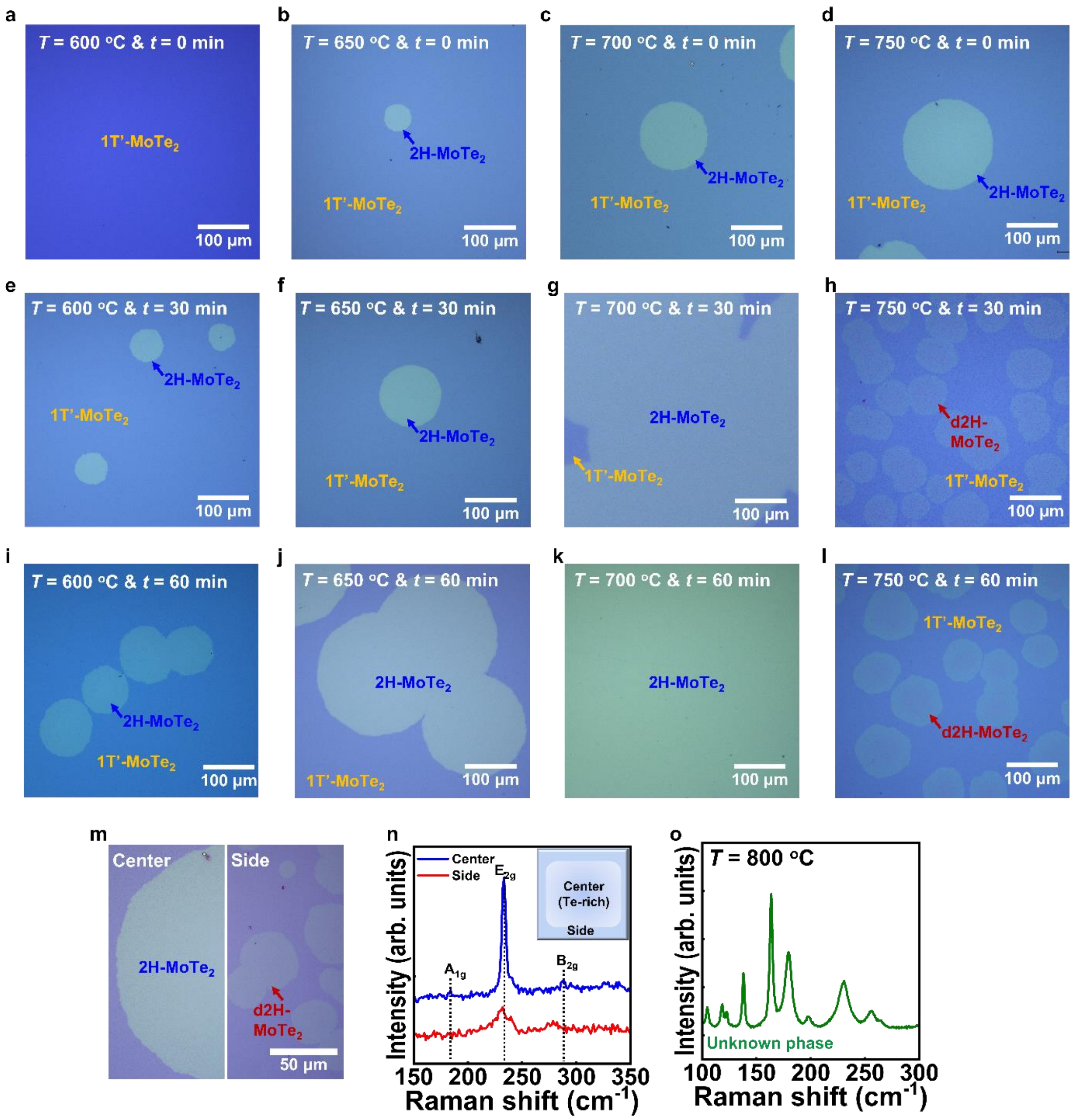

Supplementary Fig. 2. Obtained thin film varied depending on growth *T* and *t*. (**a-l**) Each *T* and *t* for the synthesis was noted in each Figure. The optical contrast difference with circular shape is the domain of 2H or defective 2H (i.e., d2H), not the 1T' $MoTe_2$. For the growth T = 700 °C and t > 60-75 min, the thin film fully covered with the 2H phase can be obtained. (**m-o**) Effect of Te-deficient conditions on the $MoTe_2$ polymorph. (**m**) Representative OM images at the center (left) and side (right) of a sample grown at T = 750 °C for t = 0 min. (**n**) Corresponding Raman spectra for the 2H-phase $MoTe_2$ depending on the locations depicted in the inset. Domains of the 2H crystals at the side of the sample exhibited vague contrast and were even smaller than those at the center, as demonstrated in the OM image in (m). Raman spectrum for the 2H phase at the side of the sample displayed that the signals from E_{2g} mode (~229 cm^{-1}) broadened significantly, indicating that the crystalline quality was worse (For an easy guide to follow, we express this crystal as defective 2H phase; shortly, d2H phase). Notably, this d2H phase is also distinguishable from the Mo_6Te_6 crystal[1], which has the characteristic Raman peaks at 156 and 249 cm^{-1}. (**o**) Representative Raman spectra for the $MoTe_2$ film grown at T = 800 °C for t = 60 min, showing the peaks for unknown structure.

: Phase transition was not successful at the side of the sample, where the Te vapor may have tended to escape from the enclosed to opened regions, resulting in the formation of the d2H phase. Furthermore, at a much higher growth temperature (T > 800°C), the 2H $MoTe_2$ crystal exhibits thermal instability, which leads to the formation of a mixed 1T'-d2H structure in our sample (Supplementary Fig. 2n). This may be attributed to the faster evaporation of Te from the Ni_xTe_y precursor at 800 °C, resulting in a Te-deficient condition that induces the formation of the 1T' structure rather than the 2H phase.

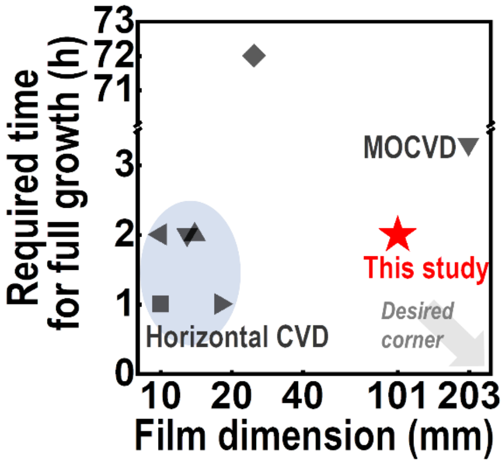

Supplementary Fig. 3. Comparison of the reported growth time to obtain the largest film dimensions in 2H-$MoTe_2$ thin film. Each symbol represents the reported parameters in ref [2-8].

: Our growth method for 2H-$MoTe_2$ has advantages over previous studies regarding processability with low energy (Supplementary Table 1). The growth time required for $MoTe_2$ across the 4-inch (~101 mm) wafer was moderate (~2 h) compared with a recent report (~72 h)[6]. The growth temperature can be decreased to 500 °C by seeding the 2H crystal, which is compatible for the direct integration process of CMOS back-end-of-line. Although MOCVD may reduce the growth temperature and time for manufacturing tri-layer $MoTe_2$[4], the resultant film was polycrystalline and exhibited inferior electrical properties; the importance of achieving high-quality crystals for a transistor is indicated in Figs. 4h, k (and Supplementary Fig. 21).

We assumed that our gas-confined growth mode enabled the production of high-quality $MoTe_2$ on a 4-inch wafer scale. The gas-confined reactor provided a high Te flux favorable for phase transition, whereas its vertical flow assisted the homogenous nucleation across the large scale. In contrast, the lateral delivery of Te flow can result in inhomogeneous random nucleation and Te deficiency during growth. However, most reports adopted the horizontal flow system, except for Refs.[4,6] in Supplementary Table 1.

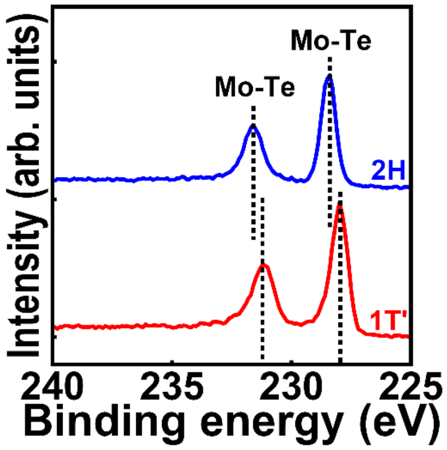

Supplementary Fig. 4. XPS characterizations for 2H- and 1T'-phase $MoTe_2$. XPS spectra of the Mo 3*d* core level captured at the 2H- (blue) and 1T'-phase crystals (red) contained at the thin films grown at *T* = 700 °C for *t* = 30 min and *T* = 500 °C for *t* = 10 min, respectively.

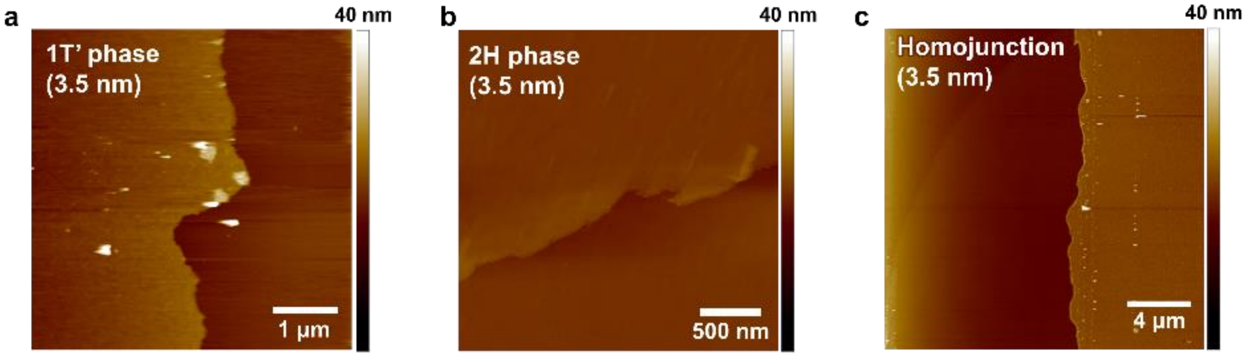

Supplementary Fig. 5. AFM images of the obtained $MoTe_2$ thin film grown using the Mo precursor. We could not find any thickness difference between the (**a**) 1T' and (**b**) 2H phase as indicated by the same *H* at homojunction (**c**), where the AFM images are captured at the region partially converted with 2H and 1T'.

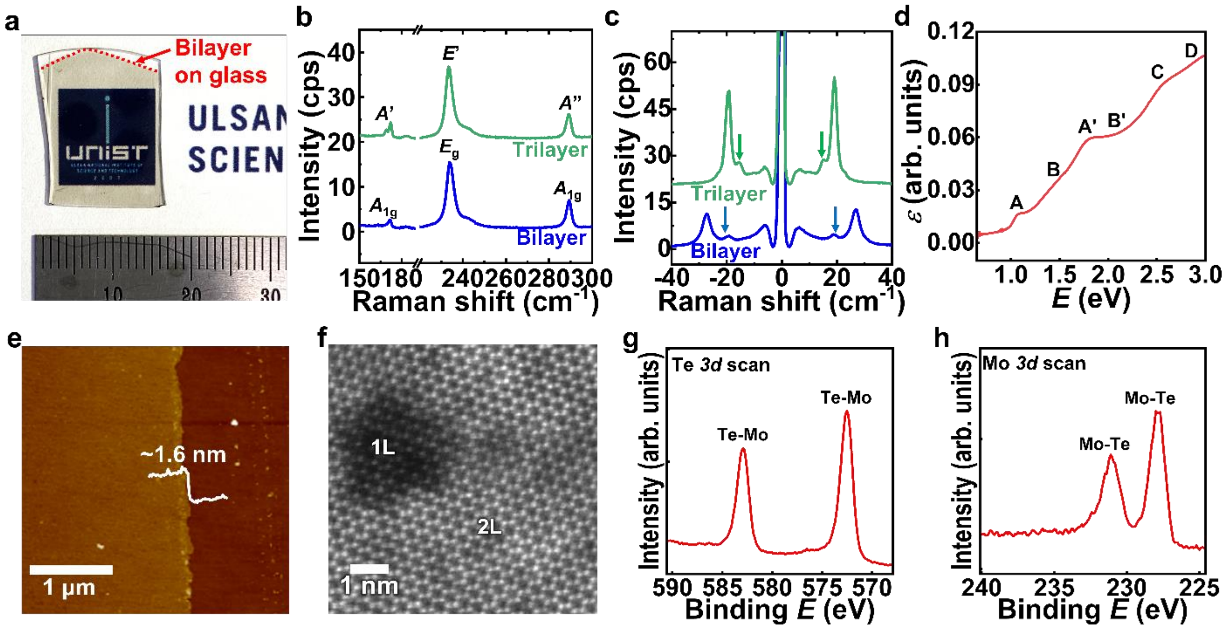

Supplementary Fig. 6. Growth of 2H-$MoTe_2$ bilayer using the MoO_x precursor. (**a**) OM image of the 2H-$MoTe_2$ bilayer grown on quartz glass with a lateral dimension of ~20 mm. (**b, c**) Raman spectra of the bilayer $MoTe_2$ film (blue) under the (**c**) high and (**d**) low frequencies of Raman shift in cm^{-1}. For comparison, the spectra of the as-grown $MoTe_2$ tri-layer film are displayed. The difference of Raman mode[9] (that is, A_{1g}, E_g, and A_{1g} in the bilayer compared to A' E' and A'' in the tri-layers), peak locations, and breath modes[10] (arrows) depending on the number of layers suggested their layer numbers. (**e**) AFM image of the bilayer thin film exhibiting its thickness (~1.6 nm). (**f**) Atomic-resolution STEM of the as-grown $MoTe_2$ structure, displaying the contrast difference for the bilayer and monolayer regions. (**g, h**) XPS of the $MoTe_2$ bilayers conducted for (**g**) Te 3*d* and (**h**) Mo 3*d* scans.

: To synthesize the 2H-$MoTe_2$ bilayer, we used MoO_x as a metal precursor instead of Mo, which allowed the atomic smoothness without void formation by decreasing the volume expansion of the precursor during the tellurization. Successful growth was suggested by the characteristic Raman modes of A_{1g}, E_g, and A_{1g} (Supplementary Fig. 6b, c)[9] and the breathing modes of few-layer structures depending on thickness[10] (arrows in Supplementary Fig. 6c). An AFM in Supplementary Fig. 6e displayed its atomically smooth surface with a thickness of ~1.6 nm and no visible micro-voids (which contradicts the growth of ~3-nm-thick film in Ref.[6]). The optical absorption spectrum of the film grown on quartz indicated that the band-to-band excitonic transition occurred at ~1.05 eV for the 2H-$MoTe_2$ bilayer (Supplementary Fig. 6d). An atomic-resolution STEM in Supplementary Fig. 6f suggested the production of $MoTe_2$ with a 2H structure. The thin film consisted of a bilayer across a large area with small monolayer domains (~2 nm in lateral dimension), but no visible holes were detected. XPS results in Supplementary Figs. 6g, h revealed the Te-Mo and Mo-Te bindings in the Te 3*d* and Mo 3*d* scans, respectively. The absence of oxide features and estimated at.% of Te/Mo (~2.01) suggested the high crystallinity of the thin film. Notably, bilayer $MoTe_2$ is the thinnest film that can be obtained using the CVD mode on a millimeter scale (Supplementary Table 1).

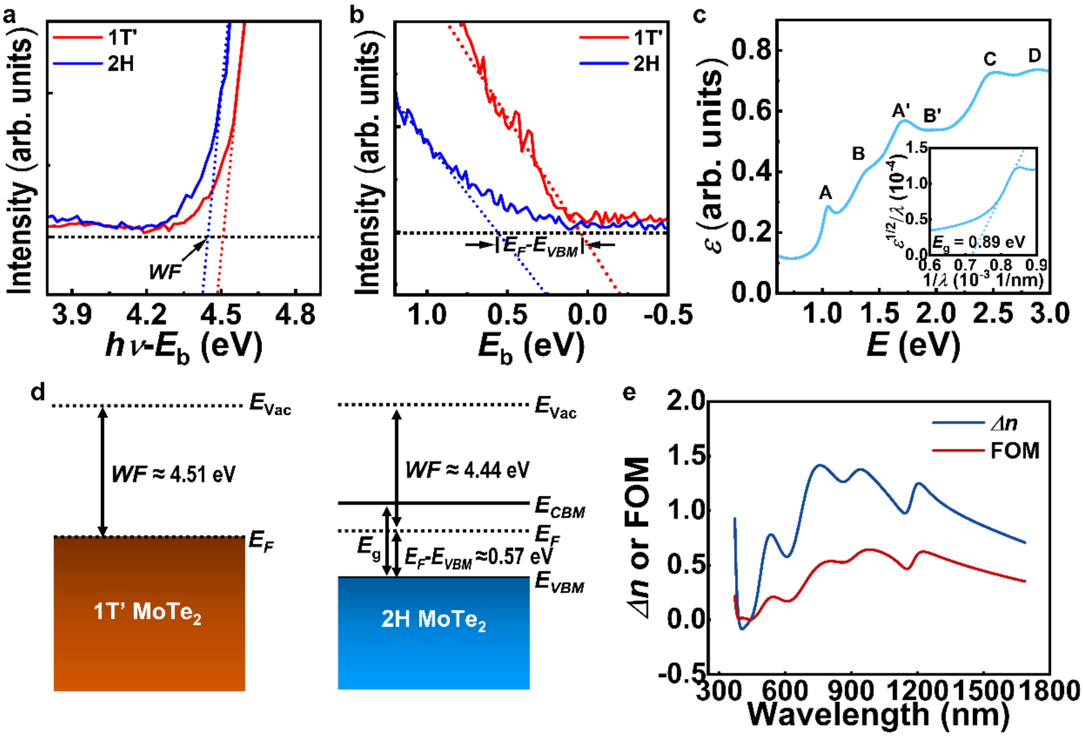

Supplementary Fig. 7. Investigation of the band structure of 1T'- and 2H-MoTe$_2$. (**a, b**) UPS spectra of 2H- (blue) and 1T'- (red) MoTe$_2$ thin film with the (**a**) high- and (**b**) low-energy regimes. Noted WF and valence band offset (E_F - E_{VBM}) indicate each extracted value at the *x*-intercept. (**c**) Absorption (ε) as a function of photon energy (E) for the synthesized 2H-MoTe$_2$ thin film. Inset shows the Tauc plot, enabling the calculation of the E_g (≈ 0.89 eV). (**d**) Schematics of the band structure of as-synthesized MoTe$_2$ polymorphs, extracted by UPS in (a) and (b). (**e**) Difference in n values between the 2H- and 1T'-MoTe$_2$ (i.e., $\Delta n = n_{2H} - n_{1T'}$) and the Figure-of-merit (FOM = $|\Delta n|/(k_{2H}+k_{1T'})$)[11] showing the potential phase-change performance.

: The measured WF of the 1T' structure was 4.51 eV, whereas WF of the 2H phase was 4.44 eV. With regards to the valance band cutoff edge (Supplementary Fig. 7a), the binding energy (E_b) of the 2H phase was 0.57 eV, corresponding to its energy level of valence band maximum (E_F - E_{VBM}). In contrast, the 1T' structure showed a sharp increase in the intensity at E_b = 0 eV owing to the filled energy levels of the metal.

To gain a better understanding of the band structure of 2H-MoTe$_2$, the ε was measured (Supplementary Fig. 7c). The peaks that signifying each transition along the Brillouin zone were labeled in the spectra[12,13]. For instance, the lowest direct optical excitonic transitions at the *K*-point (A- and B-exciton transitions)[13,14] were discovered to be at ~1.04 eV and ~1.36 eV, implying that the valance band splitting by spin-orbit coupling effect was at ~318 meV. Additionally, the E_g was calculated by following the Tauc's equation[15]; $v^2\varepsilon = (hv - E_g)^2$ where $v = 2\pi/\lambda$ is the angular frequency of the incident radiation, and λ is the wavelength. As demonstrated in the inset of Supplementary Fig. 7c, the *x*-intersection of the Tauc plot ($1/\lambda$ *vs.* $\varepsilon^{1/2}/\lambda$) indicates the $1/E_g$, and the E_g for our 2H MoTe$_2$ was extracted to be ~0.89 eV, which is in good agreement with the values obtained from the literature[12,14]. Conversely, the 1T'-phase MoTe$_2$ did not show any characteristic feature in the ε–E plot, indicating it is a gap-less material (Supplementary Fig. 7d). Thus, the appropriate phase transition of

$MoTe_2$ by our method makes it possible to engineer the band structure effectively, as depicted in Fig. 1i, satisfying the various requirements for electronic components.

It should be noted that the bandgap of our $MoTe_2$ thin film (~0.89 eV) is smaller than other group-VI TMDs, which enables $MoTe_2$ to absorb light from a broader spectral range, including the visible (VIS) and near-infrared (NIR) ranges, showing a higher absorption coefficient ($\alpha > 10^4$ cm^{-1}) (Fig. 1j). Furthermore, the high α values in our as-grown $MoTe_2$ are close to those of mechanically exfoliated single crystals[16], indicating high crystalline quality (red curves in Fig. 1j). The α value is even higher than that of bulk semiconductors with similar bandgaps, such as Si[17] or Ge[18], which are conventionally used in photodetectors. The large α values are the result of the lowest direct transition in $MoTe_2$, which has an energy close to the indirect bandgap; this is not the case for Si and Ge[19]. This suggests that $MoTe_2$ thin film has great potential for use in optical communication devices, including saturable absorbers[20], modulators[21], and photodetectors[22].

In particular, we believe that $MoTe_2$ FETs can be used as high-performance photodetectors to cover the NIR range, which cannot be achieved with other 2D TMDs with larger bandgaps. Under NIR illumination, the photoresponsivity of transistors can be enhanced by the photo-gating effect. In addition, the asymmetric contact barriers formed using different drain-source electrodes can further enhance the rectification ratio[23]. Our Fermi-level-tuned 1T'-$MoTe_2$ can serve as an efficient vdW contact for hole transport, which promises a fast photoresponse owing to fewer charge traps at the MSJ interface.

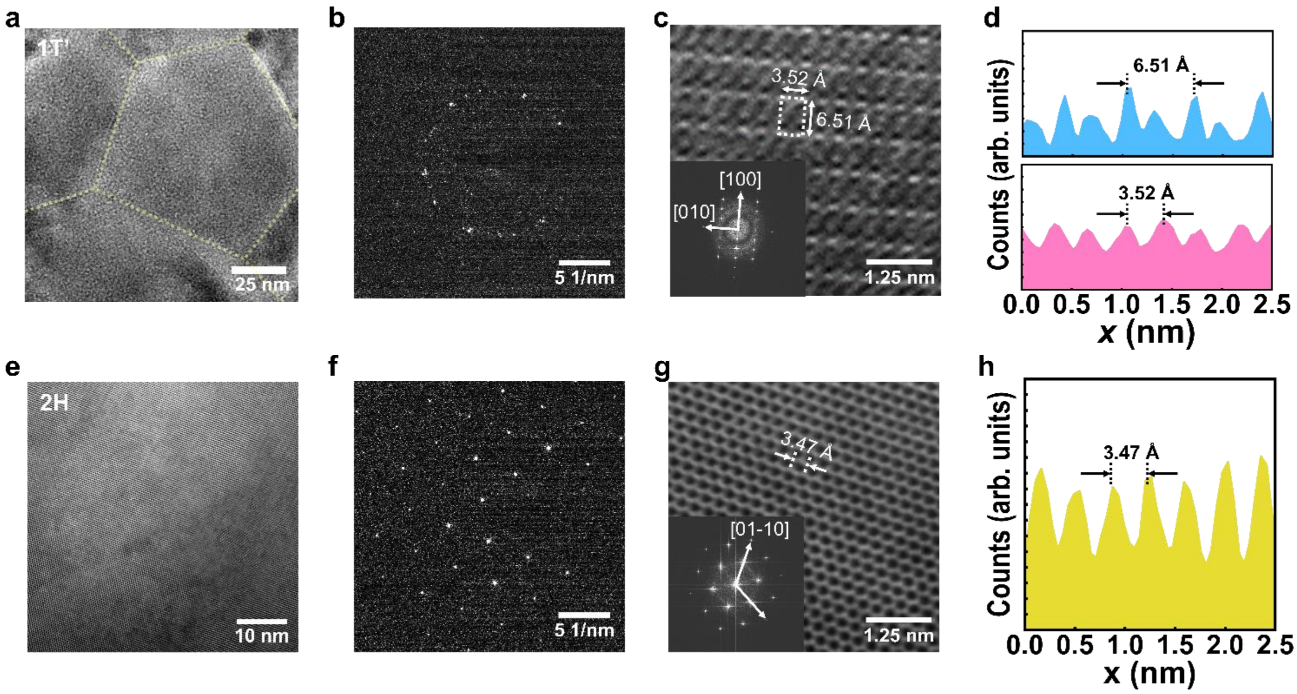

Supplementary Fig. 8. TEM analysis of the $MoTe_2$ thin film (H = 7.6 nm) with different phases. (**a–d**) TEM analysis for the 1T' phase region. (**a**) Low magnification TEM image of 1T' $MoTe_2$, showing the polycrystalline structure with a grain size of ~100 nm. Yellow lines are displayed to guide the GBs to the eyes. (**b**) Diffraction pattern of the corresponding thin film with the "ring" shapes indicating the polycrystalline nature. (**c**) High-resolution (HR)-TEM image of the 1T' $MoTe_2$ showing the unit cell of the phase. Inset shows the corresponding FFT pattern with the characteristic two-fold symmetry of the 1T' phase. (**d**) z-intensity profiles extracted from (c) displaying the repeated unit cell size. (**e–h**) TEM characterization for the 2H atomic crystals. (**e**) Low magnification TEM image of 2H $MoTe_2$ crystal without any GB-like features as in (a). (**f**) Corresponding diffraction patterns distinctively displaying each plane. (**g**) HR-TEM images of the crystal, displaying the lattice distance, extracted from (**h**) z-intensity profile.

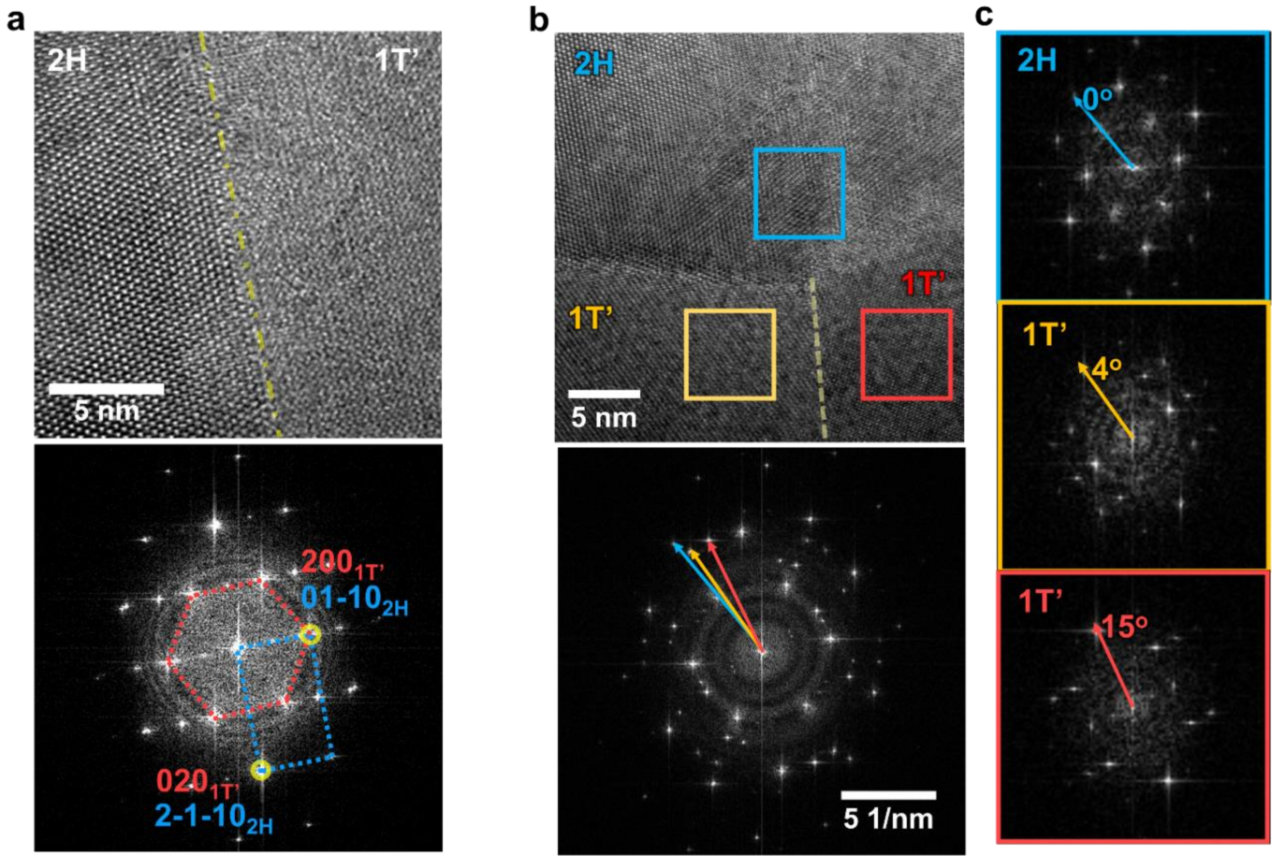

Supplementary Fig. 9. TEM analysis on the 2H-1T' interface and their crystalline orientations. (**a**, **b**) Top: HR-TEM image of the 1T' and 2H homojunction regions at a thin film. Bottom: the corresponding FFT pattern, showing the two distinct lattices of $MoTe_2$. (**c**) Extracted FFT patterns at the boxes in (**b**), showing the atomic orientation of each phase. Arrows are displayed to indicate the orientation of the plane in accordance with the 0° of FFT pattern in the 2H phase. Although the interface does not show orientation-based arrangements, the two different phases are seamlessly stitched.

: TEM analysis in (**a**) indicates that the polymorphic $MoTe_2$ crystals have a similar orientation, which can be the most favorable alignment for the grain growth of 1T' polycrystals to transform to a large 2H-phase single crystalline domain (i.e., abnormal grain growth). Conversely, many of the in-plane polymorphic interfaces demonstrated random orientations (**b, c**), which indicates that the Te-rich environment during high-temperature CVD may enable the growth of grains by overcoming the crystallographic randomness.

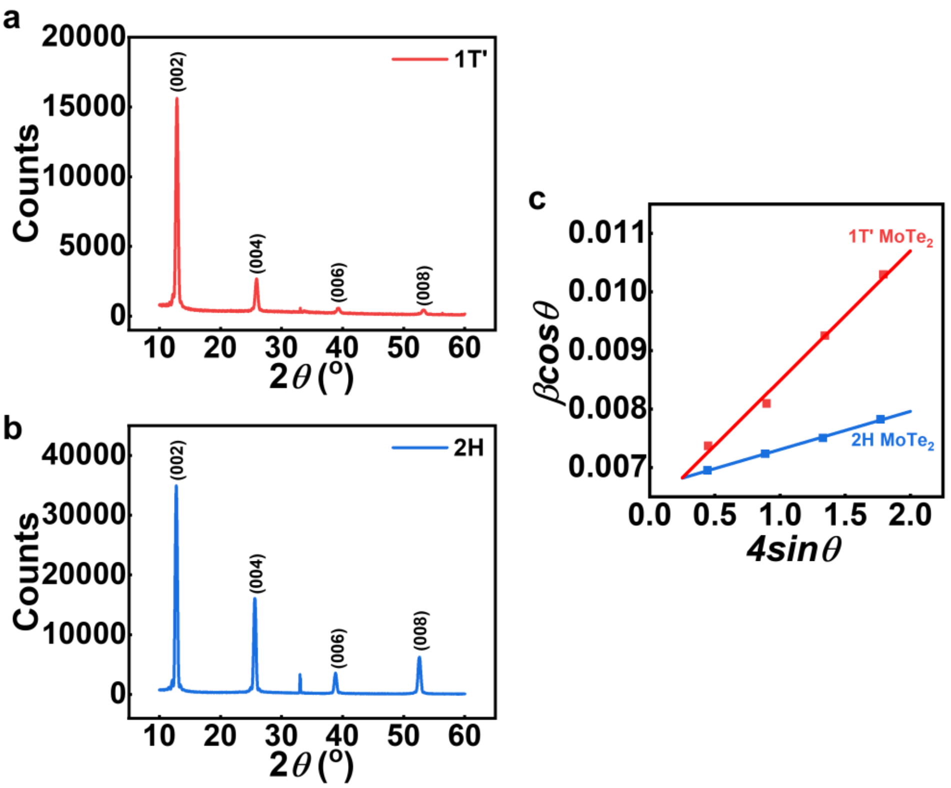

Supplementary Fig. 10. Williams–Hall method for the extraction of the microstrain of the 20 nm thick $MoTe_2$ thin film. (**a, b**) XRD patterns of the fully grown $MoTe_2$ thin film with a pure phase either (**a**) 1T' or (**b**) 2H. (**c**) Plot for the Williamson–Hall method depending on the polymorphs. The slope indicates the extracted microstrain (ε) to be ~2.22 × 10^{-3} and ~0.65 × 10^{-3} for the 1T' and 2H phases, respectively.

: The Williamson–Hall approach takes into account the broadening of the peaks as a function of a diffraction angle (2θ), which considers the combined impact of the size (β_D) and strain-driven widening (β_S) as follows:

$$\beta_S = 4\varepsilon \, tan\theta \qquad \text{(S1)}$$

$$\beta_{\mathbf{hkl}} = \beta_D + \beta_S \qquad \text{(S2)}$$

$$\beta_{\mathbf{hkl}} cos\theta = \left(\frac{k\lambda}{D}\right) + 4\varepsilon \, sin\theta \qquad \text{(S3)}$$

Hence, the extracted slopes of the Williamson–Hall plot ($4\sin\theta$-$\beta cos\theta$) in Supplementary Fig. 10c will be the ε values.

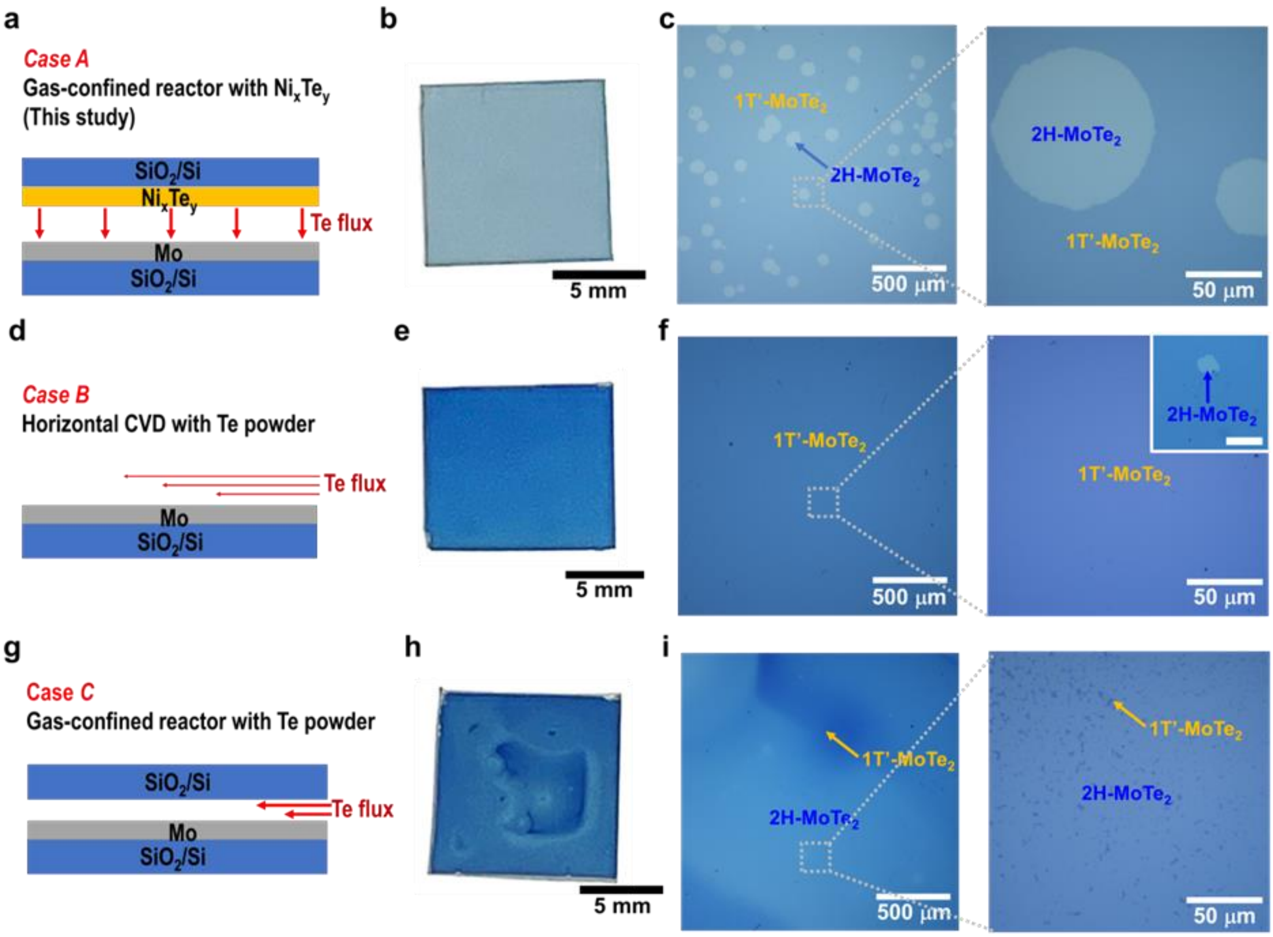

Supplementary Fig. 11. Comparison of the abnormal grain growth of the 2H phase using different growth schemes. (**a–c**) Case *A*, which involves stacked Mo and Ni_xTe_y substrates for the vertical introduction of Te (used in this study); (**d–f**) Case *B*, which is horizontal CVD using the Te powder (1 g) to introduce Te to the Mo film; and (**g–i**) Case *C*, which uses a gas-confined reactor prepared with a Mo film covered with the SiO_2/Si substrate and Te supplied using the powder (100 mg). (**a, d, and g**) Schematics of the different growth processes for Cases *A*–*C*, respectively, where the thickness of the red arrows indicates the extent of the Te flux. (**b, e, and h**) Optical images of the samples grown using (**b**) Case *A*, (**e**) *B*, and (**h**) *C*. (**c, f, and i**) Corresponding OM images taken at the center of each sample. The inset on the top right corner of (f) shows the locally transformed 2H region (scale bar: 20 μm), even though most areas of Case *B* consisted of the 1T' structure. The small dark regions in (i) indicate the local 1T'-$MoTe_2$.

: The color uniformity of the as-grown $MoTe_2$ confirmed that our method was suitable for obtaining uniform films, as shown in Case *A* (Supplementary Fig. 11b). Furthermore, in Case *A*, circular-shaped 2H domains of approximately 30–100 μm with a coverage of ~31.6% (over 1 x 1 cm^2 substrate) were evenly distributed inside the 1T' thin film, indicating abnormal grain growth (Supplementary Fig. 11c). In contrast, in Case *C*, grain growth in the 2H phase larger than several micrometers did not occur (Supplementary Fig. 11i). The 1T' and 2H phases in the thin film were intermixed, and their densities varied across the film, presumably because of the non-uniform Te flux (as seen by the ambiguous optical contrast in Supplementary Figs. 11h, i).

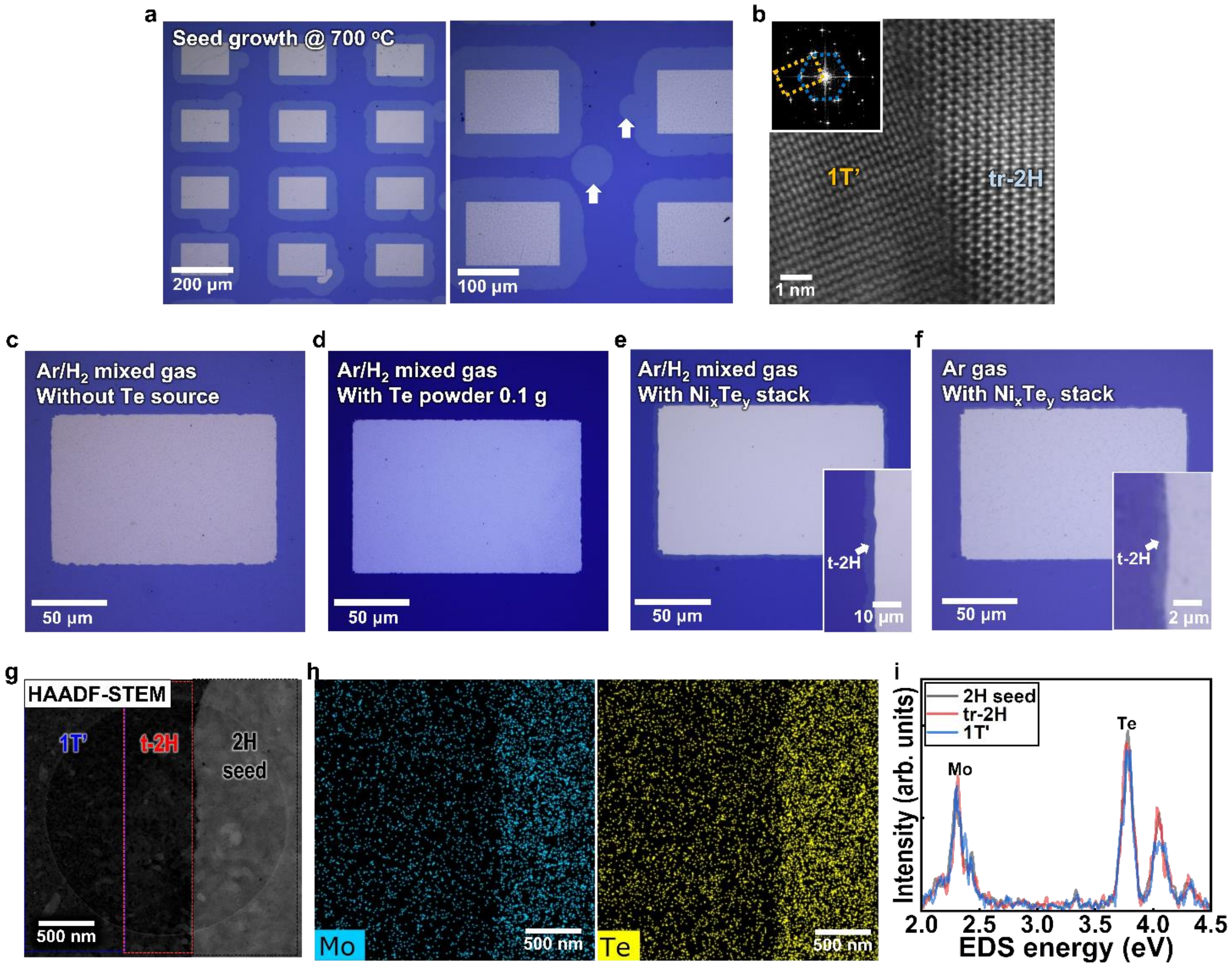

Supplementary Fig. 12. Seed growth of thin film under different ambient conditions. (**a**) OM images of the sample annealed at T = 700 °C and t = 0 min under a Te-rich atmosphere. Random 2H nucleation occurred along the thin film marked by arrows on the right. (**b**) Atomic-resolution STEM image of the 1T'/tr-2H interface region (inset: corresponding FFT patterns showing the random arrangement between the polymorphs). (**c–f**) OM images of the sample heated at T = 500 °C for t = 10 min using different carrier gases and Te sources: (**c**) without a Te source and Ar/H_2 mixed gas; (**d**) with 0.1 g of Te powder placed next to the sample and Ar/H_2 mixed gas; (**e**) using the Ni_xTe_y stack for Te-gas confinement and Ar/H_2 mixed gas; and (**f**) using the Ni_xTe_y stack and only Ar gas. The insets in (e, f) are zoomed-in OM images showing the interfaces. (**g–i**) STEM-EDS analysis of the seed growth: (**g**) low-magnified HAADF-STEM image of the structure; (**h**) corresponding EDS mapping images for Mo (left) and Te (right) atoms; and (**i**) EDS spectra for the different regions marked in (f). The stoichiometry of the structure (i.e., at.%(Te/Mo)) averaged for four different sampling regions was ~2.13 ± 0.06, 2.10 ± 0.12, and 2.02 ± 0.10 for the 2H-seed, tr-2H, and 1T' region, respectively. The sample for STEM-EDS analysis was produced by heat treatment at T = 500 °C followed by rapid cooling, i.e., furnace cover opening at T = 500 °C. Rapid cooling allows the Te adatoms to remain on the surface because there is insufficient time for the absorbed Te to be desorbed by thermal energy.

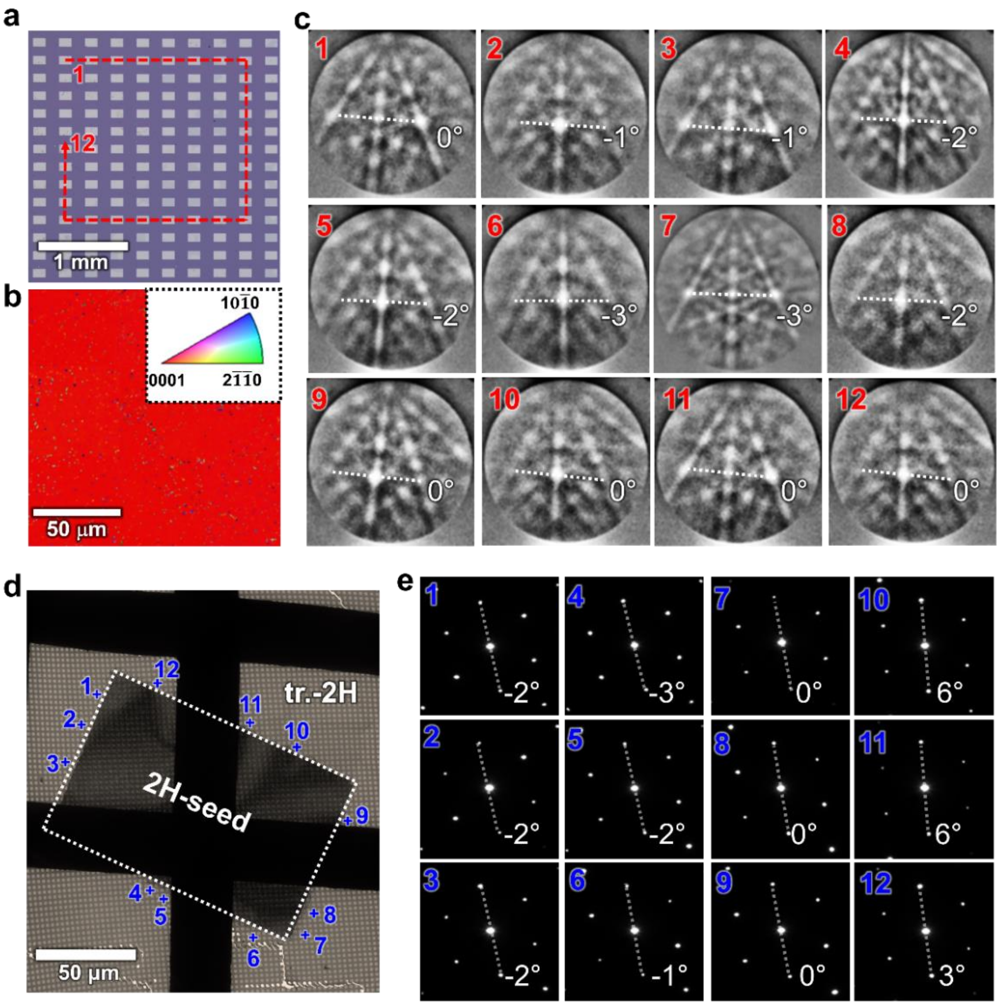

Supplementary Fig. 13. Characterizations of the large-scale single-crystalline $MoTe_2$ achieved via seed growth. (**a**–**c**) Electron backscattered diffraction (EBSD) characterization of the 2H-seed patterns with the same crystalline orientation. (**a**) OM image of 2H-seed layers on the SiO_2/Si substrate. (**b**) Representative inverse pole figure map of the 2H-seed patterns along the direction normal to surface. The uniform red color indicates the 2H-$MoTe_2$ oriented along the [0001] direction. (**c**) EBSD patterns captured for the different structures in (a), demonstrating Kikuchi patterns of $MoTe_2$ aligned in similar orientations. Measurements were taken randomly for each pattern with ~500–1,000 μm separations in the order indicated by the arrow in (a), suggesting a single-crystalline nature across an area of ~2 × 2 mm^2. (**d, e**) TEM characterization of the 2H-seed and newly transitioned area (tr-2H) of 2H-$MoTe_2$. (**d**) Low-magnified TEM image of 2H-seed (dashed rectangular) and tr-2H area surrounding the 2H-seed, transferred onto the TEM grid. (**e**) Corresponding SAED patterns captured from the tr-2H regions (positions 1–12) in (d) captured using the TEM aperture of ~40 μm. The slight difference in orientations of the patterns for the 10–12 regions may be attributed to the bending of the film induced by the TEM grid.

: In this study, the initial seed layers were made of mechanically exfoliated single-crystalline flakes. The "bulk" $MoTe_2$ mother crystal (obtained from HQ graphene) had a single crystalline property over a large area (> ~5 mm). Different flakes transferred on the tape had the same crystal orientation when adhesive tape was simultaneously applied and removed from the crystal. This tape/flake sample was then transferred onto a preformed 1T'-$MoTe_2$ film (with a thickness of 20 nm), followed by seed growth and conversion to the 2H phase at 500°C. The low-temperature growth at 500 °C allowed the suppression of random 2H nucleation during the seed growth process. The resulting fully grown 2H film was single-crystal in nature along the unidirectional crystalline orientation of the

exfoliated seeds (at least across an area of ~2 × 2 mm^2, as shown in Supplementary Figs. 13a-c). The seeded-grown 2H-flakes/film structure could be patterned using photolithography for subsequent seed growth to synthesize unidirectionally oriented single crystals, as shown in the OM image (Supplementary Fig. 13a). Inverse pole figure mapping (Supplementary Fig. 13b) and EBSD (Supplementary Fig. 13c) patterns confirmed that the conformally formed patterns were single crystals with (0001) texture and the same alignment or thin films with a small-angle GB (with a standard deviation of ~1.19°). Moreover, TEM analysis showed that the newly grown "tr.-2H" regions were all oriented in a similar direction, indicating the single-crystalline nature (Supplementary Figs. 13d, e).

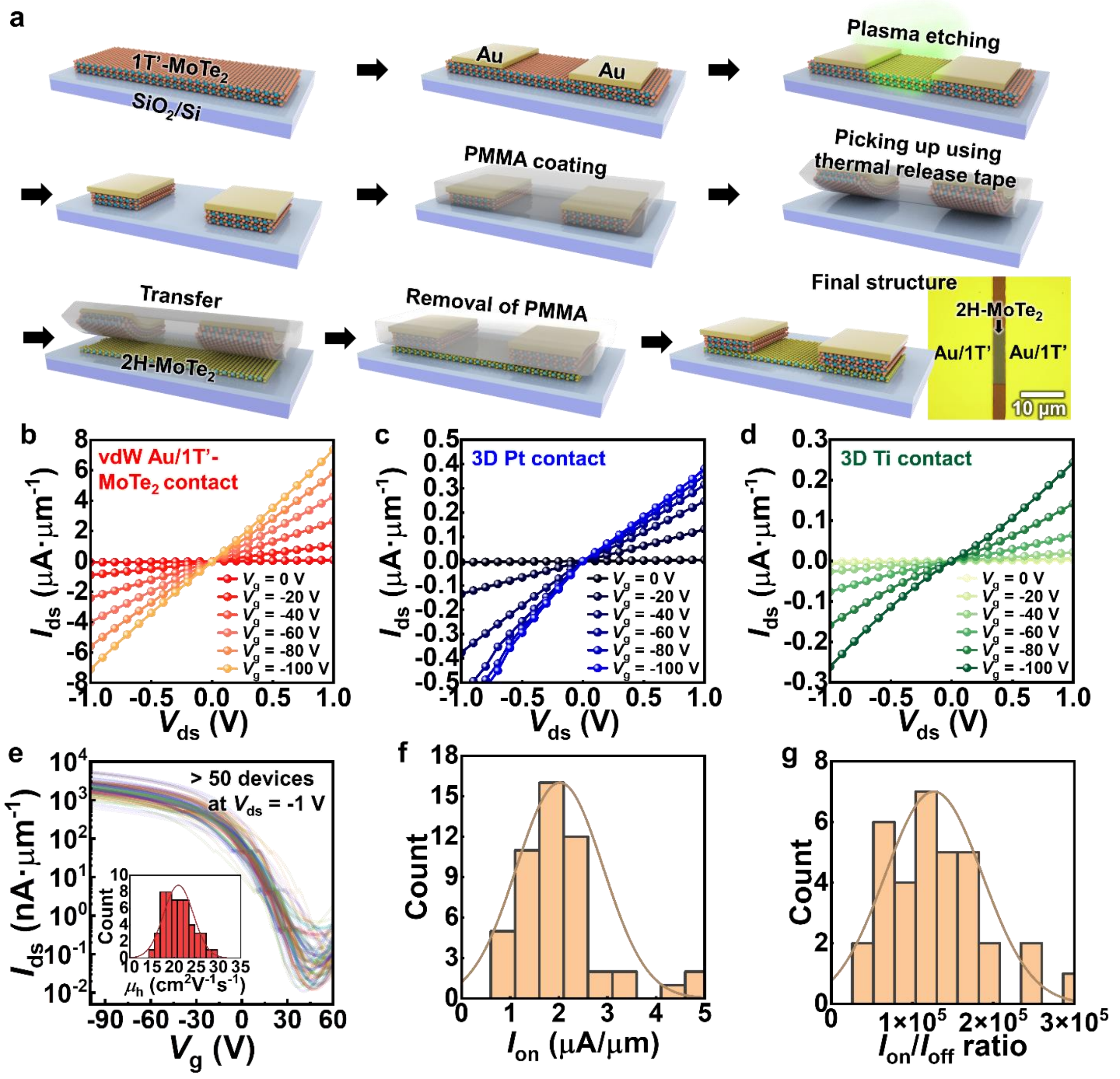

Supplementary Fig. 14. Fabrication and characterizations of the vdW-integrated 1T'-$MoTe_2$/2H-$MoTe_2$ junction FET arrays. (**a**) Schematic depicting the fabrication of the $MoTe_2$-based transistor. At first, the synthesis was conducted at the different growth T and t for a specific phase; for example, T = 700 °C, t = 60 min, and T = 500 °C, t = 30 min for the 2H and 1T' structure, respectively. To achieve the Au/1T'-$MoTe_2$ patterns, the arrays of Au layers (~40 nm) were deposited using standard lithography and an e-beam evaporator, and then the reactive ion etching (RIE) process removed the exposed 1T'-$MoTe_2$, except for the underlying structure below the Au patterns. Next, the dry transfer process using the thermal release tape and the polymeric supporting layer of PMMA allowed the achievement of the Au/1T'-$MoTe_2$/2H-$MoTe_2$ junction. The RIE procedure was followed for the definition of channel width for FETs. The inset on the right corner shows a top-view OM image of a fabricated FET with a 2H-$MoTe_2$ channel. (**b**–**d**) Room-temperature I_{ds}-V_{ds} output characteristics of 2H-$MoTe_2$ FETs with (**b**) vdW Au/1T'-$MoTe_2$ semimetal, (**c**) 3D Pt and (**d**) Ti contact electrodes. (**e**–**g**) Electrical measurements of fabricated FET arrays on a chip. (**e**) I_{ds} *vs*. V_g for the representative sample of 50 devices with an L of ~5–9 μm on a chip measured at V_{ds} = -1 V. Inset shows the histogram demonstrating the μ_h values of the 50 different FETs with 1T'-$MoTe_2$ contact. (**f**–**g**) Histogram representing the (**f**) I_{on} and (**g**) I_{on}/I_{off} values of the 50 different FETs with Au/1T'-$MoTe_2$ contact.

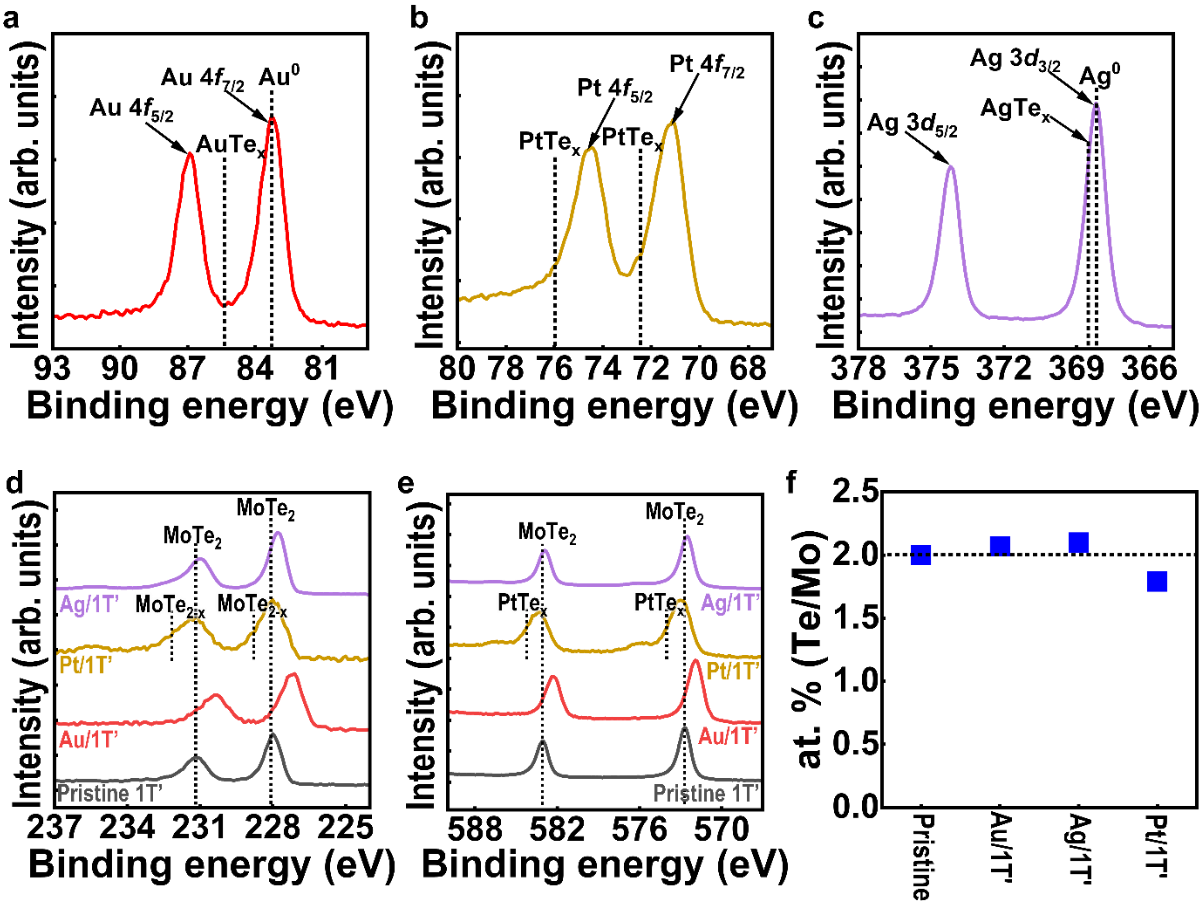

Supplementary Fig. 15. 3D metals deposited onto the vdW 1T'-$MoTe_2$ semimetal and their interfacial interactions. (**a**–**e**) XPS spectra of the 1T'-$MoTe_2$ sample with the deposited Au (red), Pt (gold), and Ag (purple) for the scans of (**a**) Au 4*f*, (**b**) Pt 4*f*, (**c**) Ag 3*d*, (**d**) Mo 3*d*, and (**e**) Te 3*d* core levels. XPS for the pristine 1T'-$MoTe_2$ is also demonstrated as grey curves. Peak positions related to the non-stoichiometric Mo_xTe_y, intermetallic $PtTe_x$, and pristine $MoTe_2$ are displayed as dashed lines in (d, e) as a guide for the eyes. We could not identify the formation of $AuTe_x$ or $AgTe_x$ intermetals in the XPS. (**f**) Comparison of the $MoTe_2$ stoichiometries, showing a large deviation from the ideal value [i.e., at.%(Te/Mo) = 2] in the Pt-deposited FET.

: Au/1T'-$MoTe_2$ and Ag/1T'-$MoTe_2$ sample peaks exhibit shifts towards lower binding energy compared to those of the pristine 1T'-$MoTe_2$, which is unrelated to any stoichiometric change (Supplementary Fig. 15f) and the chemical interaction between 3D metals and Te. Instead, we believe that the high-carrier-density 3D metal deposition on the 1T'-structured semimetal impacts its electronic properties, shifting the Fermi level by charge transport (i.e., doping). For instance, the UPS-driven WF of the Au/1T'-$MoTe_2$ was ~5.0 eV, showing the deviation from the as-grown layer with a WF of ~4.45 eV (Fig. 5b).

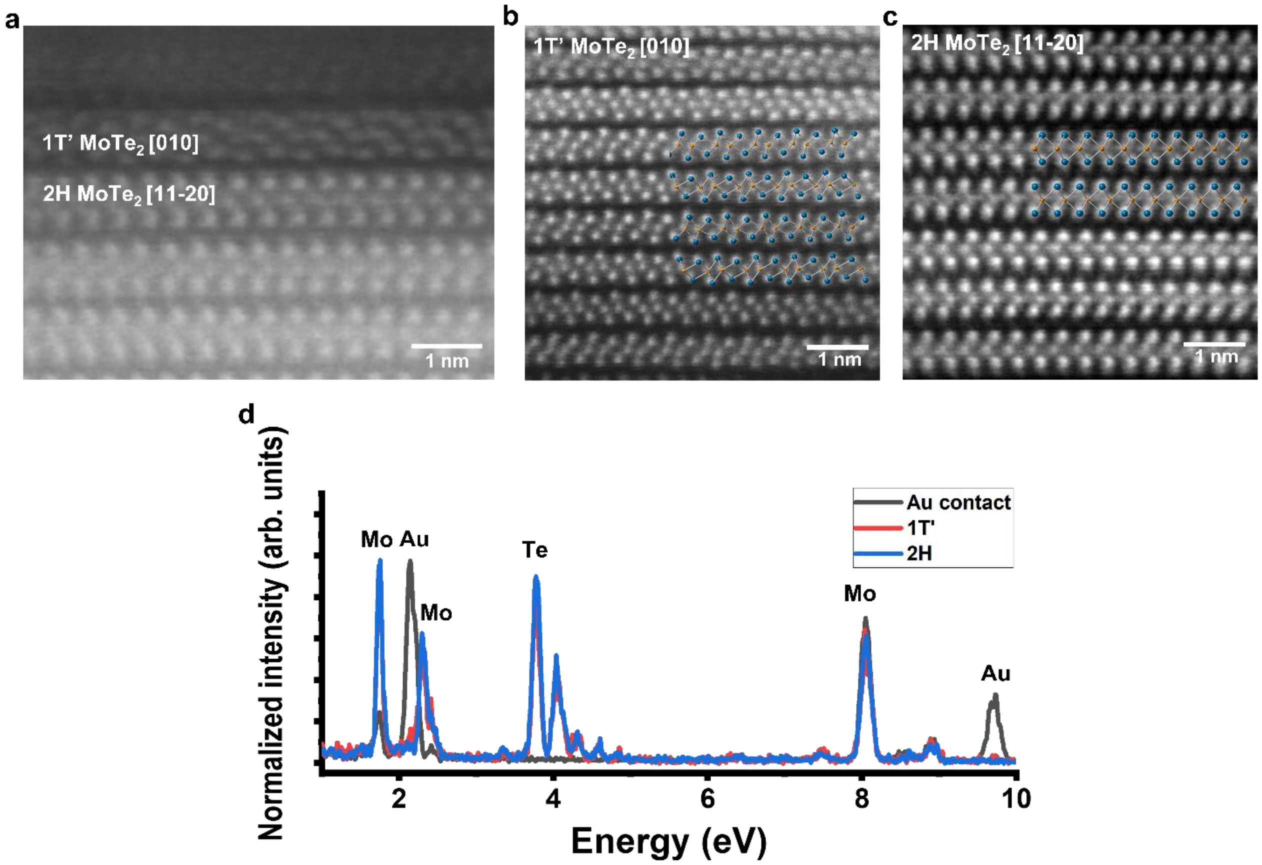

Supplementary Fig. 16. TEM analysis of the fabricated 1T'/2H-$MoTe_2$ heterojunction. (**a**) Atomic-resolution STEM image of the 1T'-$MoTe_2$/2H-$MoTe_2$ interface. (**b**, **c**) Atomic-resolution STEM images of each structure of $MoTe_2$ captured after fabricating the heterostructure. (**d**) Representative EDS spectra for each material, i.e., 1T'-$MoTe_2$ (red), 2H-$MoTe_2$ (blue), and Au contact (black). The 2H- and 1T'-$MoTe_2$ showed at.%(Te/Mo) of ~2.14 and 2.11, respectively, indicating no Te vacancy formation during the fabrication of the heterojunction.

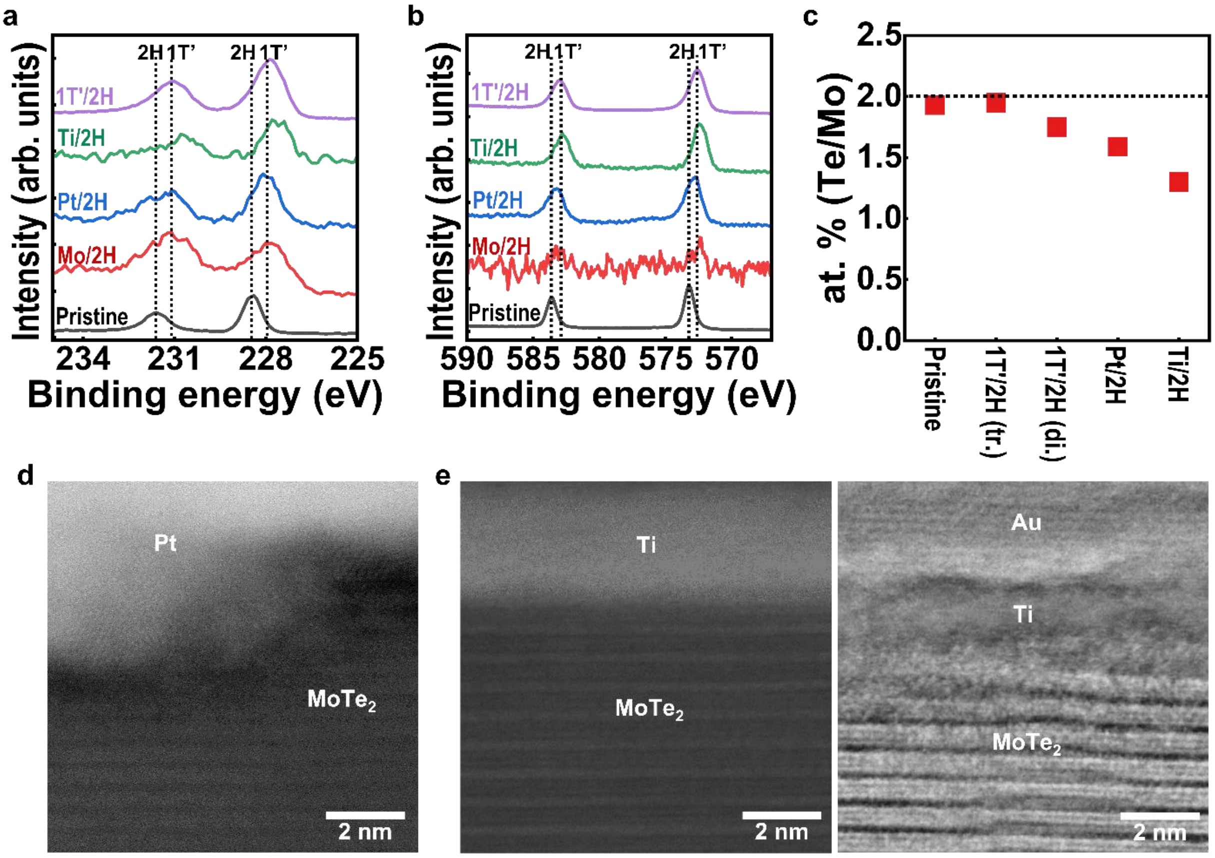

Supplementary Fig. 17. Characterization of the interface between 3D metal and 2H-MoTe₂. (**a, b**) XPS spectra of the (semi-)metal–semiconductor junction, fabricated using metal deposition (i.e., Ti/2H, Pt/2H, and Mo/2H) or the transfer of 1T'-$MoTe_2$ (i.e., 1T'/2H) on 2H-$MoTe_2$. XPS spectra of the as-grown 2H-$MoTe_2$ surface have been shown for comparison. (**c**) Summary of the stoichiometry of the 2H-$MoTe_2$ junctions, characterized by XPS. For the 1T'-$MoTe_2$/2H-$MoTe_2$ structures, we could also fabricate using a direct synthesis of 1T'-$MoTe_2$ [1T'/2H (di.)] by the Mo deposition onto 2H-$MoTe_2$ followed by its tellurization. However, the synthesis results in the non-stoichiometric 1T'/2H $MoTe_2$ junctions, which show a substantial difference from the one created by the transfer method we devised [1T'/2H (tr.)]. (see Supplementary Fig. 14 for more details) (**d, e**) Cross-sectional STEM images of (**d**) Pt/2H-$MoTe_2$ and (**e**) Au/Ti/2H-$MoTe_2$ junctions. Both samples show metallic extrusion. Because of the strong chemical interactions, layer distortion was prevalent at the Ti/$MoTe_2$ interface, but in Pt, defects in the form of breaking and penetrating the layers were more common.

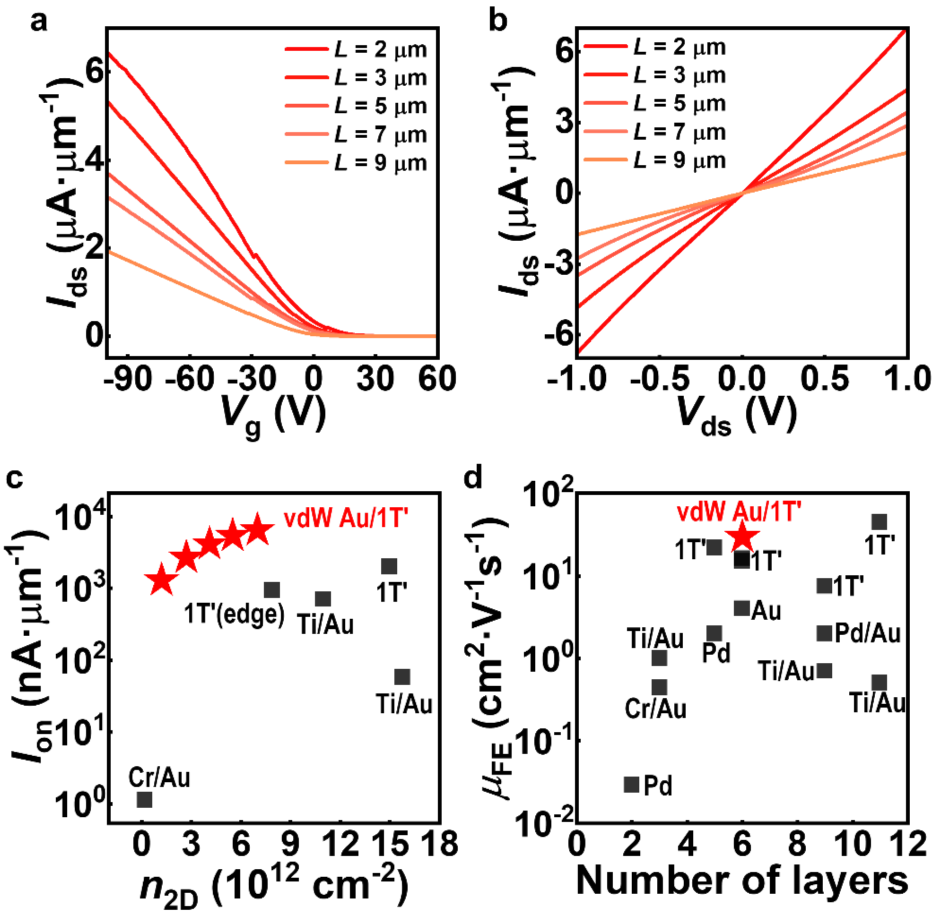

Supplementary Fig. 18. Evolution of transport behavior in polymorphic $MoTe_2$ transistors as a function of channel lengths and layer numbers. (**a, b**) Electrical transport of 2H-$MoTe_2$ FETs with different channel L (2–9 μm) at RT, whose carriers were injected from the Fermi-level-tuned vdW Au/1T'-$MoTe_2$ contacts. (**a**) Representative transfer (I_{ds}–V_g) curves measured at V_{ds} = -1 V, and (**b**) corresponding output characteristics (I_{ds}–V_{ds}) under a V_g of –100 V. (**c**) Extracted I_{on} of 2H-$MoTe_2$ transistors with vdW Au/1T' contacts and their comparisons with various literatures depending on the n_{2D}. (**d**) Comparisons of field-effect hole mobilities (μ_{FE}) in the 2H-$MoTe_2$ transistors depending on the number of layers. The different contact electrodes used in Refs.[2,3,5-7,24-33] are labeled.

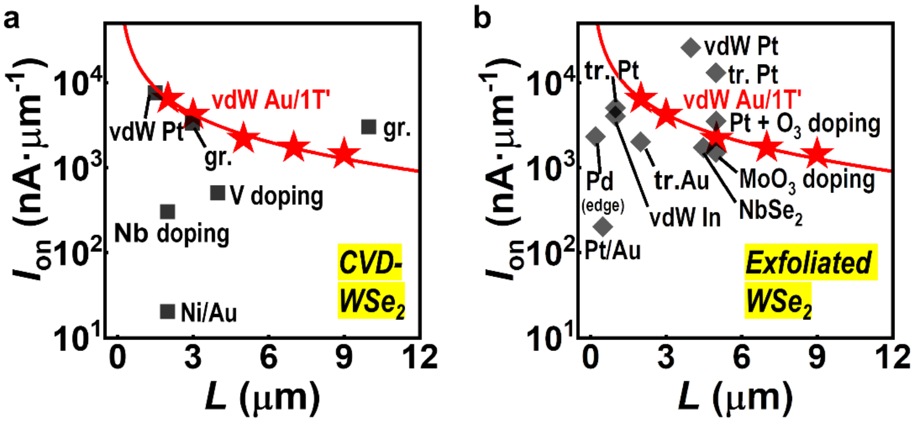

Supplementary Fig. 19. Comparison of I_{on} values (at V_{ds} = -1 V) of synthetic 2H/1T'-$MoTe_2$ transistors in this study with reports for *p*-type (**a**) CVD-grown[23,34-39] and (**b**) mechanically exfoliated WSe_2[23,40-48] dependent on channel *L*. Our data were fitted with the reciprocal relationship between I_{ds} and *L* (i.e., $I_{ds} \propto 1/L$). The substitutional[34,35] or oxidation-related doping[41,42] methods or used contact metals (e.g., graphene (gr.)[37,38], the transferred (tr.) metals[43-46], and the cleanly deposited metals with vdW gap (vdW metals)[23,40]) are noted (see Supplementary Table 4 for more comparisons). Most of the WSe_2 transistors reported here were based on the irregularly synthesized or mechanically exfoliated flakes, except MOCVD[39] and this study.

: The challenge in preparing a superior *p*-type device is contradictory to the technological maturity for the construction of *n*-type 2D transistors for MoS_2, particularly compared to the cases using low-melting-point metals such as Bi[49] and In[40] (note that I_{on} of MoS_2 approached 1,135 μA·μm^{-1} in Ref.[49]). For example, WSe_2 is a promising candidate as a unipolar *p*-type channel owing to its relatively high valence band edge compared with MoS_2 and WS_2. However, most CVD-grown WSe_2 still exhibited the I_{on} values of 0.02–3.30 μA·μm^{-1} under the V_{ds} of -1 V[34-39] even after its doping[34,35] or employing 2D metal contact electrodes (e.g., graphene)[37,38], which is more than two times lower than that of our synthetic $MoTe_2$ polymorphic transistor (~7.8 ± 1.4 μA·μm^{-1}) (see Supplementary Fig. 19a and Supplementary Table 4 for the comparisons). Furthermore, the calculated R_c in CVD-WSe_2 (16.3–10^5 kΩ·μm)[23,34,37,38] was higher than that obtained in this study (Supplementary Table 4). Note that the similar I_{on} of ~7.6 μA· μm^{-1} in a CVD-WSe_2 flake could be achieved by the ultraclean Pt contact[23], but the deposition process required a longer period (~4 h) to reduce the irradiation energy. Although one study proved that the synthetic vertical heterostructure of VSe_2/WSe_2 allowed the large I_{on} of ~1,580 μA· μm^{-1} in an ultrashort channel (~20 nm)[50], the process is still embryonic in terms of device manufacturing in any desired channel dimension using standard lithographic techniques. Nearly all CVD-WSe_2 transistors were constructed based on tiny irregular flakes rather than a wafer-scale film[23,34-38,50] (owing to the limitation of scalability), raising the question of reproducibility and device-to-device variations.

The I_{on} of our *p*-type $MoTe_2$ transistor was also comparable to or even higher than those reported for high-performance devices manufactured for mechanically exfoliated WSe_2[23,40-48] (Supplementary Fig. 19b and Supplementary Table 4). Compared to the thicker WSe_2 transistors (5–9 layers[41,42,44,47,48] or unidentified[23,40,45,46]), the superior I_{on} reported in our study indicates that $MoTe_2$ can also be an outstanding candidate for *p*-type 2D transistors when the high-quality channel and defect-free contact are combined. In addition, the valence band edge of the $MoTe_2$ higher than that of WSe_2[51] allows more unipolar *p*-type transport in a transistor, which has the advantage of achieving lower power consumption and faster operation in the CMOS inverter.

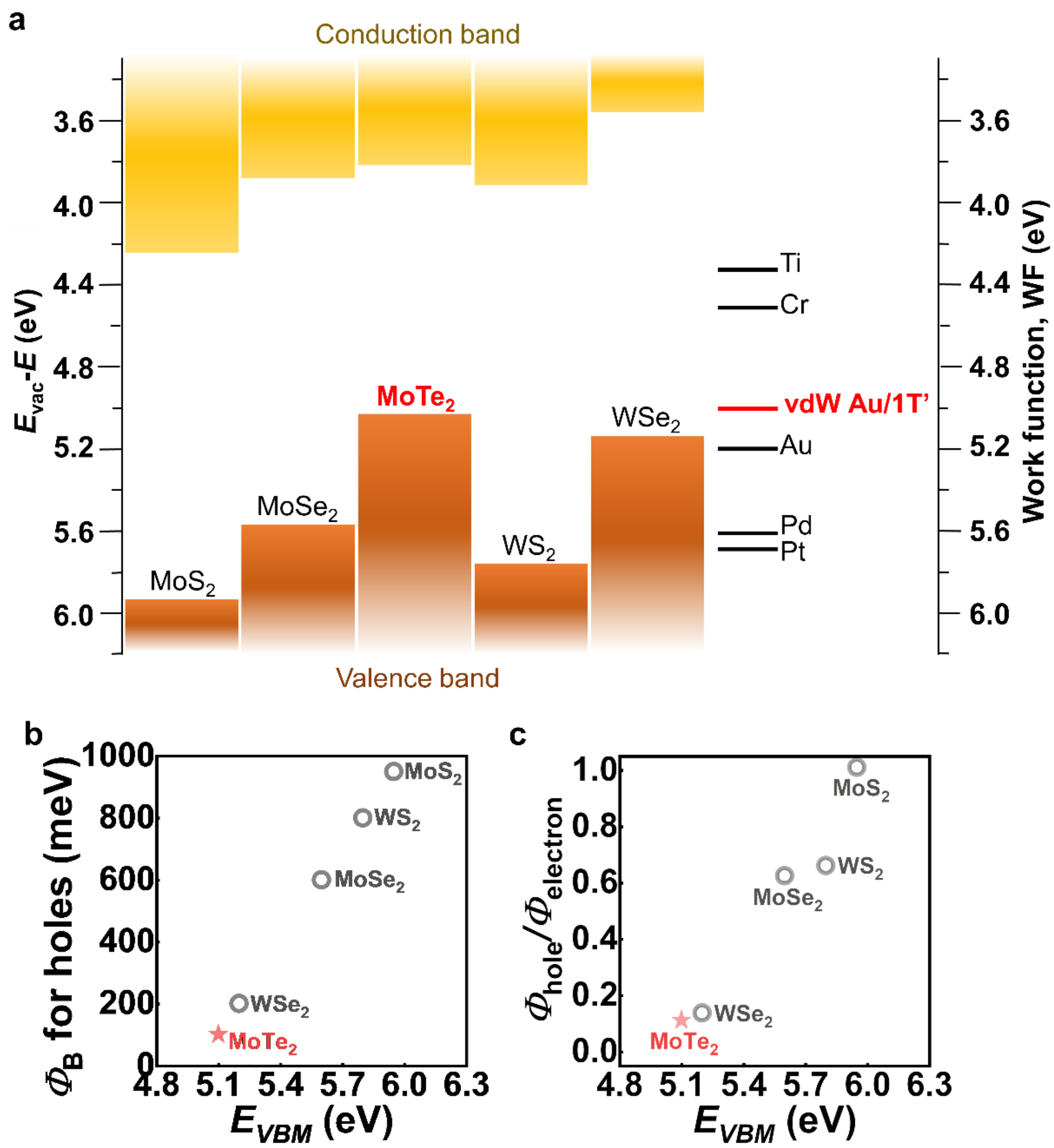

Supplementary Fig. 20. Estimation of hole injection efficiency depending on the 2D group-VI TMDs. (**a**) (Left) Band structures of group VI-TMDs[52], and (right) WFs of conventional 3D metals (black) and vdW Au/1T'-$MoTe_2$ (red) used in this study. (**b**) Calculated thermionic barrier height (Φ_B) for holes at the flat band voltage, depending on the valence band maximum (E_{VBM}) of each 2D semiconducting TMD. The estimation assumed the ideal Schottky-Mott model for metal contact with a WF of ~5.0 eV. (**c**) The ratio between the Φ_B values for holes and electrons ($\Phi_{hole}/\Phi_{electron}$) as a function of E_{VBM}.

: $MoTe_2$ can behave as a better *p*-type channel than other group-VI 2D TMDs in CMOS, given its band structure. Typical group-VI 2D TMDs other than 2H-$MoTe_2$ present a challenge in suppressing electron transport owing to their E_{VBM} edge located at > 5.0 eV (Supplementary Fig. 20a). This results in a high SBH for holes (Φ_{hole} at V_{FB}) in these 2D TMDs, which limits the hole conductivity (Supplementary Fig. 20b). In contrast, $MoTe_2$ has a lower SBH owing to its higher location of E_{VBM}, making its hole conductivity higher (Supplementary Fig. 20b). Notably, the ratio between Φ_{hole} and $\Phi_{electron}$ of $MoTe_2$ is also the smallest among the group-VI TMDs (Supplementary Fig. 20c). Consequently, $MoTe_2$-based 2D FETs tends to exhibit *p*-type unipolarity (instead of ambipolarity), promising a low power-delay product per bit in CMOS owing to the smaller off-state currents in *p*-type MOS and greater efficiency in high-low and low-high transitions.

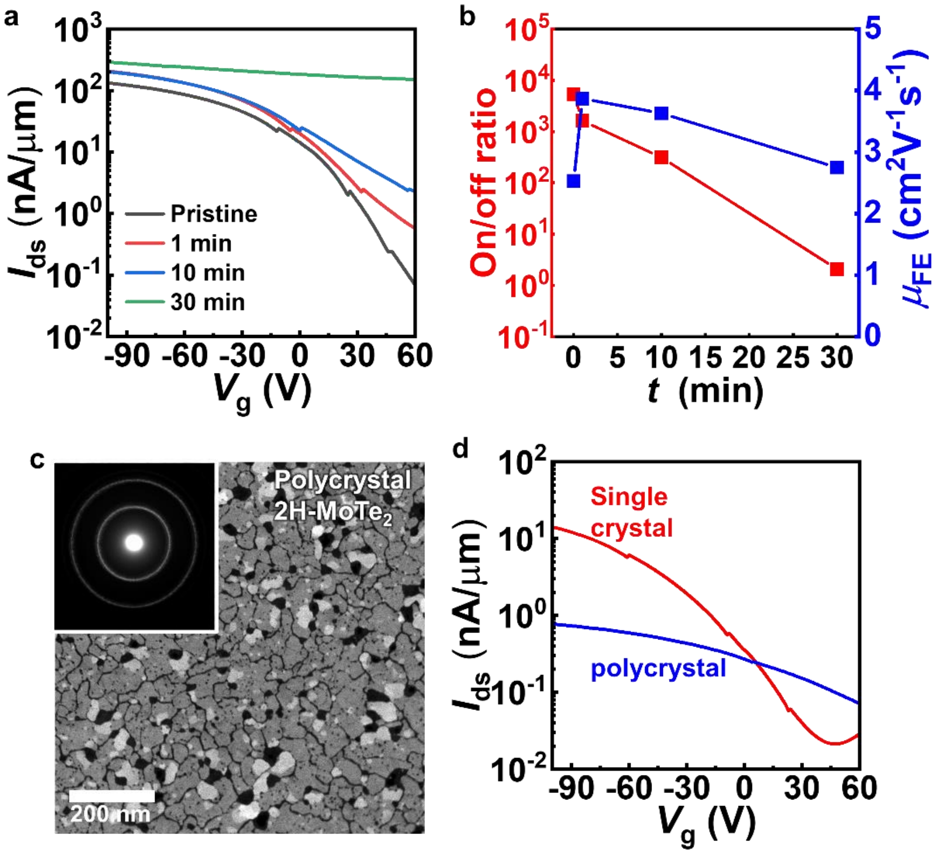

Supplementary Fig. 21. Effect of oxidation and GBs on the electrical transport of 2H-$MoTe_2$ FETs with 3D metal contact electrodes. (**a, b**) Oxidation-induced *p*-type doping of a 2H-$MoTe_2$ FET with Pt contact electrodes. (**a**) Transfer curve (I_{ds}–V_g) of the FET depending on the oxidation time (e.g., 1, 10, and 30 min). Here, the oxidation process[53] was conducted using a commercial ultraviolet (UV)-O_3 cleaning system (Ahtech, AC-3). (**b**) Evolution of the on/off ratio and μ as a function of the oxidation time (t). Oxidation was observed to degrade switching behavior by decreasing the on/off ratio as the I_{off} gets higher. Numerous studies on 2H-$MoTe_2$-based FETs show a small on/off ratio (< 10^3) despite its high μ and low sheet resistance, which could probably be affected by the oxidation, as $MoTe_2$ is vulnerable to oxidation more than other semiconducting TMDs. (**c, d**) Polycrystalline 2H-$MoTe_2$ thin film and its electrical transport. The polycrystalline 2H-$MoTe_2$ thin film was obtained via suppression of the abnormal grain growth mode of 2H-$MoTe_2$ by controlling the Te flux. (**c**) Representative TEM image of the 2H-$MoTe_2$ thin film displaying small poly-grains of ~30–50 nm. The inset shows the SAED patterns of the polycrystalline thin film with a ring-type diffractogram. (**d**) Transfer characteristics of FETs based on 2H-$MoTe_2$ single crystals (red curve) and polycrystalline channels (blue curve) in contact with Ti/Au electrodes measured at V_{ds} = -1 V. The reduction of I_{on} in the polycrystalline 2H-$MoTe_2$ FET is possibly due to GB scattering.

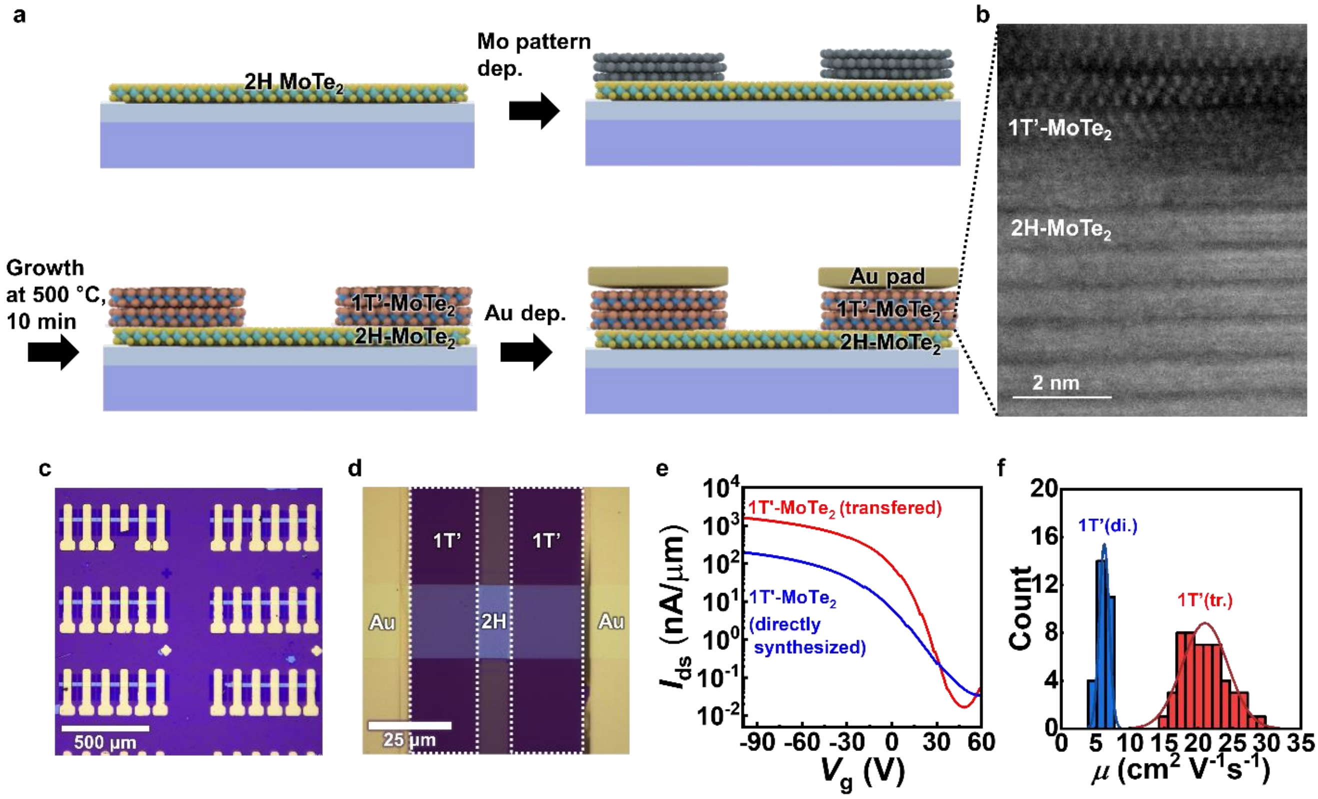

Supplementary Fig. 22. FETs based on the directly synthesized 1T'-$MoTe_2$/2H-$MoTe_2$ heterojunction with the amorphous and non-stoichiometric layers. (**a**) Schematics showing the synthesis-based fabrication of the vertical 1T'-$MoTe_2$/2H-$MoTe_2$ heterostructure. (**b**) Representative STEM image showing the cross-section of the synthesized junction. (**c, d**) OM images of the fabricated devices on SiO_2/Si substrate. Zoom-in image of (**d**) shows each composed material in the FETs. (**e**) Transfer curves of the FETs with directly synthesized 1T'-$MoTe_2$ contact. To compare the carrier transport behavior, the current values obtained from the counterpart that was fabricated using the transfer process is demonstrated as a red curve. (**f**) Distribution of the μ_h values of the 1T'/2H-$MoTe_2$ heterostructure FETs, formed either using the direct synthesis (i.e., 1T' (di.); blue) or dry transfer (i.e., 1T' (tr.); red).

: The direct synthesis method for fabricating 2D/2D polymorphic heterojunction resulted in a poor FET performance in comparison to the transfer process we suggested in the main text (Supplementary Fig. 14). Contact properties could be degraded due to the amorphous structure (Supplementary Fig. 22b) and non-stoichiometric $MoTe_2$ (Supplementary Fig. 17c). We believe that the direct deposition of Mo on top of as-grown 2H-$MoTe_2$ using DC sputtering could produce some deformed phases, as we observed new peaks for 1T'-like structures in the XPS of the Mo/2H-$MoTe_2$ (Supplementary Figs. 17a, b). Nevertheless, there are some viable options for producing vdW contacts using the synthesis method instead of transfer, where the process includes the vertical growth of TMDs without any interfacial defects[50,54-56]. We believe that the low-energy deposition of MoO_x or MoI_2 as precursors for 1T'-$MoTe_2$ is a possible solution for preparing ultraclean vdW contact electrodes in a synthetic manner devoid of performance degradations[50,54]. The use of thermally degradable buffer layers[55,56] is another option for realizing vdW 1T' $MoTe_2$ contacts.

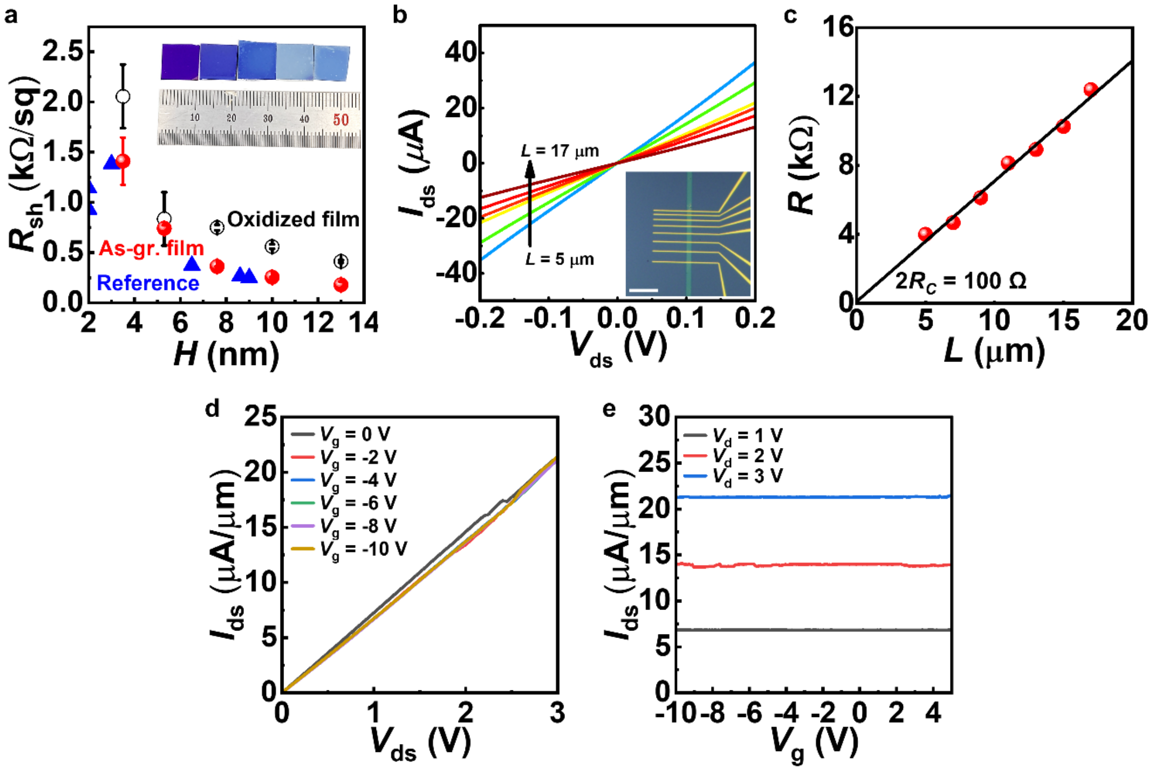

Supplementary Fig. 23. Electrical characterization of the as-synthesized 1T'-$MoTe_2$. (**a**) Sheet resistance (R_{sh}) of the large-area 1T' $MoTe_2$ thin film (~1 × 1 cm^2) characterized by the four-point measurement. As-grown sample (red) demonstrated an R_{sh} lower than the $MoTe_2$, which was exposed to air for ~1 h (black). For comparison, the R_{sh} of a single-crystalline $MoTe_2$ flake[57] is demonstrated in blue. The data points represent the average ± standard deviation of five different large-area samples for each set. (**b**) I_{ds}–V_{ds} characterizations of the TLM device with 1T'-$MoTe_2$ channel contacted to the Ti/Au with an L of 5–17 μm. Inset shows the OM image of the fabricated 1T'-$MoTe_2$ device with a TLM pattern (scale bar: 25 μm). (**c**) Corresponding TLM plot, showing a low R_c of 51 Ω (~0.51 kΩ·μm) and a low R_{sh} of ~7.0 kΩ·sq^{-1}. (**d**, **e**) V_g dependence of the electrical transport of the FET with an active layer of 1T'-$MoTe_2$ (H = 15 nm), i.e., (**d**) output and (**e**) transfer characteristics.

: The 1T'-$MoTe_2$ thin film was characterized using the four-point probe and TLM (Supplementary Fig. 23). The method offered significant control over the H (≈3.5–13 nm) and enabled the systematic characterization of the H-dependent R_{sh} for a large-area thin film (> 1 × 1 cm^2) via four-probe measurement (Supplementary Fig. 23a). The R_{sh} values were comparable to those of single-crystalline 1T' $MoTe_2$[57], indicating that the crystals are of high quality. The R_{sh} was increased as the layer got thinner, owing to the enhanced carrier scattering. As a result of the position-controlled growth method of $MoTe_2$ (see the method), the width-defined layer with H of ~5.3 nm was simply contacted to the Ti/Au TLM patterns via conventional photolithography and deposition approaches (Supplementary Fig. 23b-inset). I_{ds}–V_{ds} curve illustrates the linear relations with a channel length dependence, which is typical evidence for ohmic contacted TLM devices (Supplementary Fig. 23b). R_{sh} (~7.0 kΩ·sq^{-1}, which is comparable to the TLM-extracted values for mechanically exfoliated single crystals[58,59]) and R_c (≈ 0.51 kΩ·cm) values of the 1T'-$MoTe_2$ device were determined using the linear fit to the L-dependent R (Supplementary Fig. 23c). Notably, the R_c was at the low end of the reported 3D metal/2D metal systems values[60]. Additionally, the 1T'-$MoTe_2$ showed a negligible dependence on the V_g (Supplementary Fig. 23d, e), which is a characteristic feature of a gap-less electrical conductor.

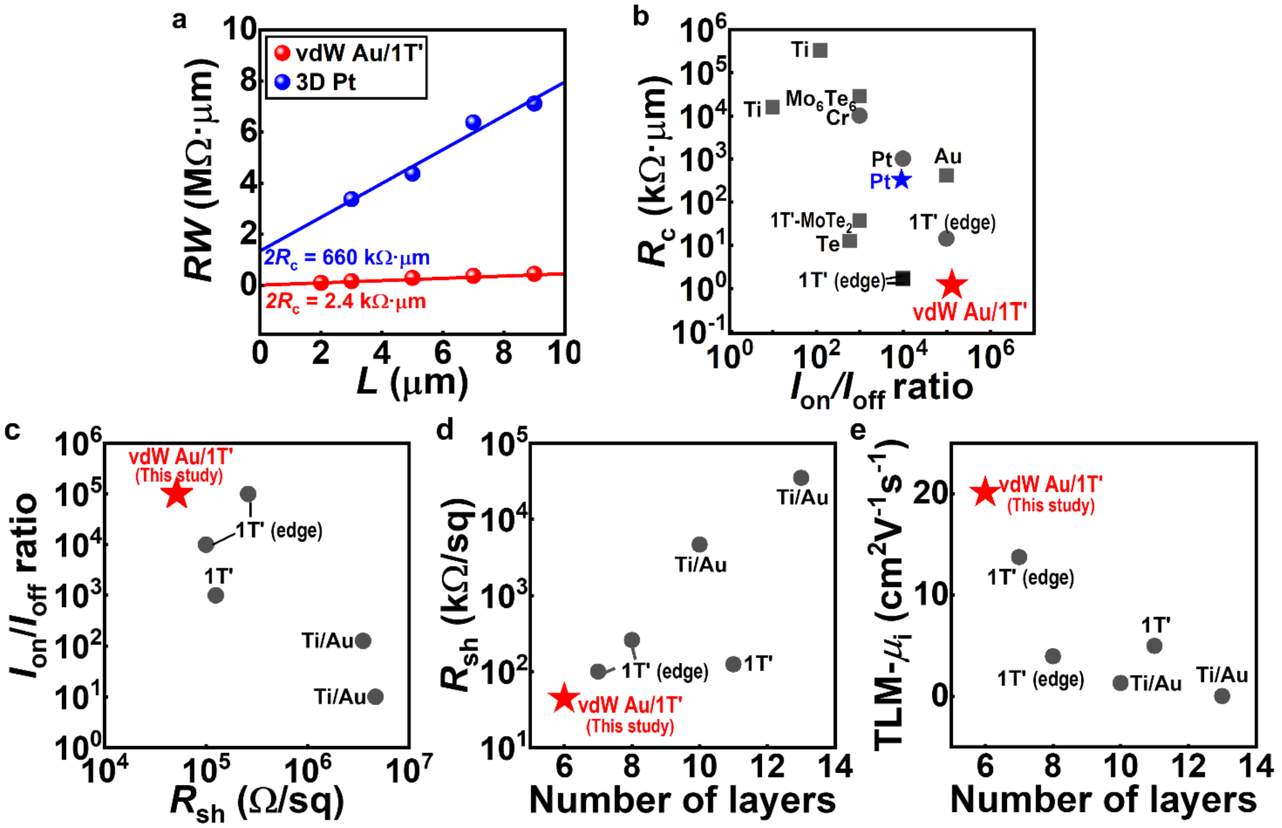

Supplementary Fig. 24. Electrical characterization of 2H-$MoTe_2$ FETs with vdW Au/1T'-$MoTe_2$ and 3D Pt contact electrodes consisting of the TLM patterns. (**a**) TLM plots at V_g = -100 V (which corresponds to the induced n_{2D} of ~7.5-7.9 × 10^{12} cm^{-2}) showing the RW depending on the L averaged for more than five TLM sets on 2H-$MoTe_2$ FETs with vdW Au/1T'-$MoTe_2$ and 3D Pt contact electrodes. (**b**) Comparison of R_c and I_{on}/I_{off} obtained in this study with those reported previously for 2H-$MoTe_2$ FETs[3,5,6,24,25,27,61-64]. (**c**) I_{on}/I_{off} ratio and R_{sh} obtained using TLM patterns in our 2H-$MoTe_2$ FETs, compared to previous reports[3,5,24,25,27]. (**d**) Comparison of on-state R_{sh} values as a function of the number of 2H-$MoTe_2$ layers according to literature[3,5,24,25,27]. (**e**) "Intrinsic" field-effect mobility (μ_i) extracted using TLM measurements plotted against the layer numbers in previous reports for 2H-$MoTe_2$[3,5,24,25,27].

: We utilized the TLM approach to determine the on-state R_{sh}, estimated to be ~44.3 ± 2.3 kΩ/sq at V_g = -100 V (as observed from the slopes of TLM curves in Fig. 4i and Supplementary Fig. 24a). The on-state R_{sh} value represents a "material-dependent" quantity and does not incorporate contributions from device dimensions and contact resistance. Our analysis of on-state R_{sh} of 2H-$MoTe_2$ indicates that our synthesis method results in the lowest R_{sh} (44.3 ± 2.3 kΩ/sq), the highest I_{on}/I_{off} ratio (> 2.9 × 10^5), and the smallest layer numbers (~6 layers), compared to those of TLM-analyzed CVD-grown $MoTe_2$ FETs in previous studies[3,5,24,25,27] (Supplementary Fig. 24c, d).

Furthermore, the relationship between the on-state R_{sh} (~44.3 ± 2.3 kΩ/sq) obtained through TLM and the V_g-induced carrier concentration (n_{2D} ~7 × 10^{12} cm^{-2}) permits us to determine the "intrinsic" field-effect mobility (μ_i) of our $MoTe_2$ using the following relationship[65-67]:

$$\mu_i = \frac{1}{qR_{sh}n_{2D}} \qquad \text{(S4)}$$

The calculated value of μ_i is ~20.2 ± 1.1 $cm^2V^{-1}s^{-1}$, representing an inherent channel property that is unaffected by contact resistance. Notably, this value closely matches the averaged two-terminal field-effect mobility (μ_h ≈21.0 ± 3.3 $cm^2V^{-1}s^{-1}$; inset of Supplementary Fig. 14e), indicating that our 2D semimetal contact electrodes have a minimal impact on μ_h. Moreover, the TLM-extracted μ_i value in our study (~20.2 ± 1.1 $cm^2V^{-1}s^{-1}$) surpasses the calculated values for CVD-grown $MoTe_2$ in previous studies[3,5,24,25,27] (Supplementary Fig. 24e; from the reports[3,5,24,25,27], the on-state R_{sh} value is extracted from the slopes of TLM plot, and n_{2D} is obtained using a parallel capacitance model as $n_{2D} = C_{ox}(V_g - V_{th})/q$.). Given that all the compared FETs in Refs.[3,5,24,25,27] utilize a bottom-gate SiO_2 dielectric layer, the μ_i or n_{2D} values are primarily influenced by material properties such as defect density and doping capacity, rather than the device configuration or dielectric interface.

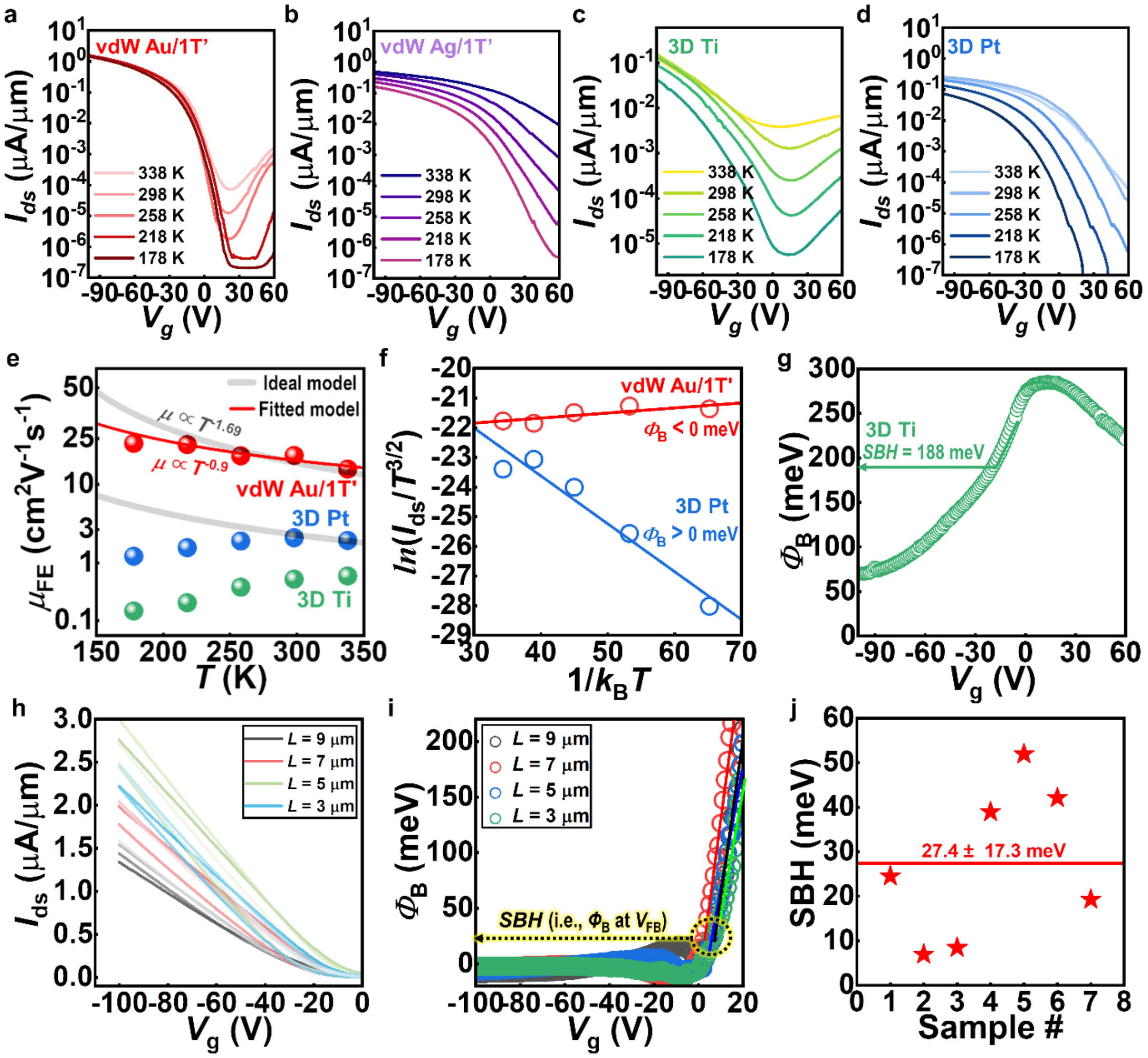

Supplementary Fig. 25. Electrical transport of 2H-MoTe$_2$ FETs with vdW semimetal and 3D metal contacts at various temperatures. (**a**–**d**) Typical I_{ds}–V_g of 2H-MoTe$_2$ MSJ FETs with (**a**) vdW Au/1T'-MoTe$_2$, (**b**) vdW Ag/1T'-MoTe$_2$, (**c**) 3D Ti, and (**d**) 3D Pt contact electrodes at various temperatures. (**e**) Two-terminal hole mobilities of 2H-MoTe$_2$ FETs with vdW Au/1T'-MoTe$_2$ (red), 3D Ti (green), and 3D Pt (blue) contact electrodes as a function of temperature. The temperature dependence of the mobility, $\mu \propto T^{-\gamma}$, indicates the dominant phonon scattering of the FET. (**f**) Arrhenius plots of the vdW Au/1T'/2H-MoTe$_2$ (red) and Pt/2H-MoTe$_2$ (blue) MSJ FETs. The extracted Φ_B values are 152.2 meV and -1.6 meV for Pt/2H-MoTe$_2$ and 1T'/2H-MoTe$_2$ FETs at n_{2D} of ~6 × 10^{12} cm^{-2} ($\Delta V_g \approx 1$ V), respectively. (**g**) Φ_B values extracted at various V_g for hole transfer of a 3D Ti-contacted 2H-MoTe$_2$ FET. The Φ_B value at the flat band voltage was 188 meV. (**h**–**j**) Reproducibility of SBH for the 2H-MoTe$_2$ FET with vdW Au/1T'-MoTe$_2$ contact electrodes in TLM patterns. (**h**) I_{ds}-V_g curves measured at different T (~218–338 K) for FETs with different channels L (~3–9 μm). What looks more pronounced in the same color indicates that it was measured at a higher T. (**i**) Corresponding Φ_B of the 1T'/2H-MoTe$_2$ FETs, which exhibit similar behavior regardless of the L. The curves demonstrate the linearity of the Φ_B before reaching the V_{FB}, at which the true SBH can be extracted. (**j**) The calculated SBH of eight different devices with an average value of 27.4 ± 17.3 meV.

: The gray lines for a power law ($\mu_{FE} \propto T^{-\gamma}$ where γ = 1.69) in Supplementary Fig. 25e indicate the ideal phonon scattering model from the theoretical calculation[68]. For our 2H-$MoTe_2$ with vdW Au/1T' contact, the new damping factor, γ, with T > 218 K was positive (0.92 ± 0.24 (mean ± standard error)), indicating that the transport was still limited by phonon scattering[69]. The slight deviation from the ideal value (~1.69) could arise due to the T-dependence of the effective Schottky barrier height[69,70] and/or interplay between homopolar phonon mode quenching and charge-impurity scattering[71]. In contrast, the 2H-$MoTe_2$ FETs with 3D metal contacts (i.e., Pt and Ti) exhibited a decrease in μ_{FE} as T decreased, indicating that transport was constrained by the contact resistance rather than phonon scattering.

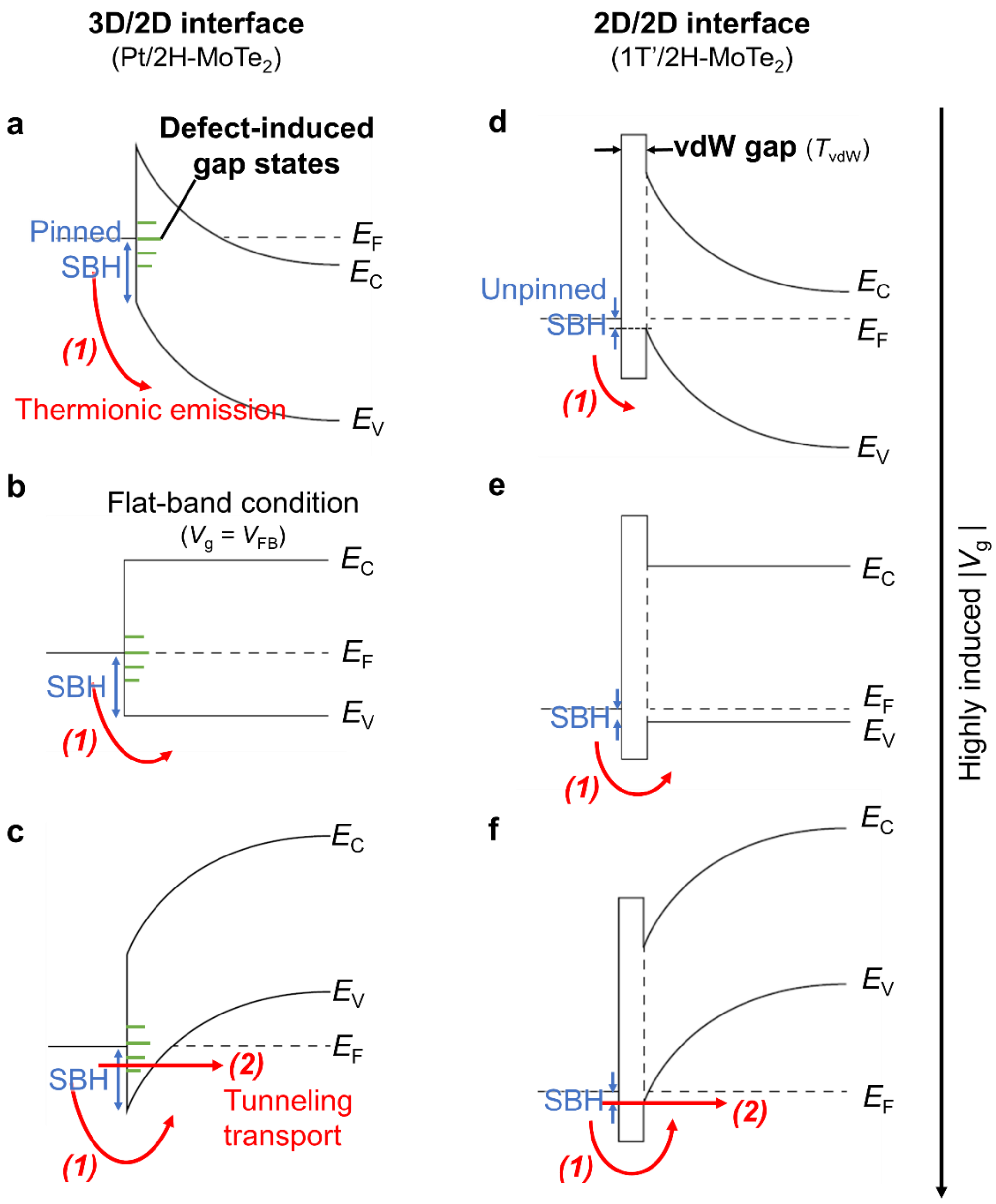

Supplementary Fig. 26. Band diagrams depicting the charge transport mechanism. Hole injection through the Schottky barrier for (**a**-**c**) 3D/2D MSJ of Pt/2H-$MoTe_2$, and (**d**-**f**) the 2D/2D interface at 1T'/2H-$MoTe_2$ is demonstrated based on the *p*-doping of the channel layer by decreasing V_g (or largely applied V_g down to –100 V), e.g., (**a**, **d**) $V_g > V_{FB}$, (**b**, **e**) $V_g = V_{FB}$, and (**c**, **f**) $V_g < V_{FB}$. (*1*) and (*2*) signify thermionic emission and tunneling transport, respectively. Although the WF values of Pt (~5.6 eV) and Au/1T'-$MoTe_2$ (~5.0 eV) are high enough to obtain negligible SBH, the extracted SBH of Pt/2H-$MoTe_2$ MSJ (~174 meV) is higher than that of 1T'/2H-$MoTe_2$ MSJ (~27.4 meV) owing to the pinned Fermi level at the interface states caused by defects between Pt and 2H-$MoTe_2$ (i.e., atomic discontinuity, microscopic defects or clusters of phases, and other effects as discovered in Supplementary Fig. 17).

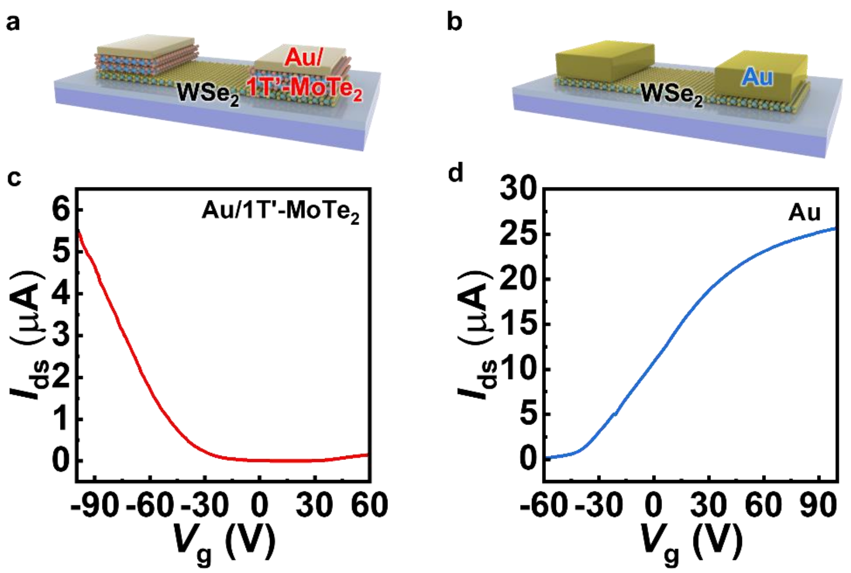

Supplementary Fig. 27. Universality of our vdW semimetal contact for *p*-type atomic transistors. (**a, b**) Schematic of the WSe_2 FETs in contact with the (**a**) vdW metal (i.e., Au/1T'-$MoTe_2$ stack) and (**b**) 3D metal (i.e., Au). (**c, d**) Representative transfer characteristics (I_{ds}-V_g) of the WSe_2 MSJ FETs at $|V_{ds}|$ = 1 V, exhibiting the (**c**) *p*-type dominant characteristic by the vdW Au/1T'-$MoTe_2$ contact and (**d**) *n*-type behavior by the evaporated Au contact. The mechanically exfoliated multilayer WSe_2 was used as a channel for the devices to avoid doping effects observed in the monolayer or CVD-grown ones. Given the similar WFs of the metal contacts (~5.0–5.1 eV; Fig. 5f), it is evident that the pristine vdW contact effectively induced the MSJ with a small SBH for holes by following the Schottky–Mott rule. In contrast, the high-energy deposition of Au stimulates the formation of the gap state near the conduction band[43,72], resulting in the *n*-type transport behavior.

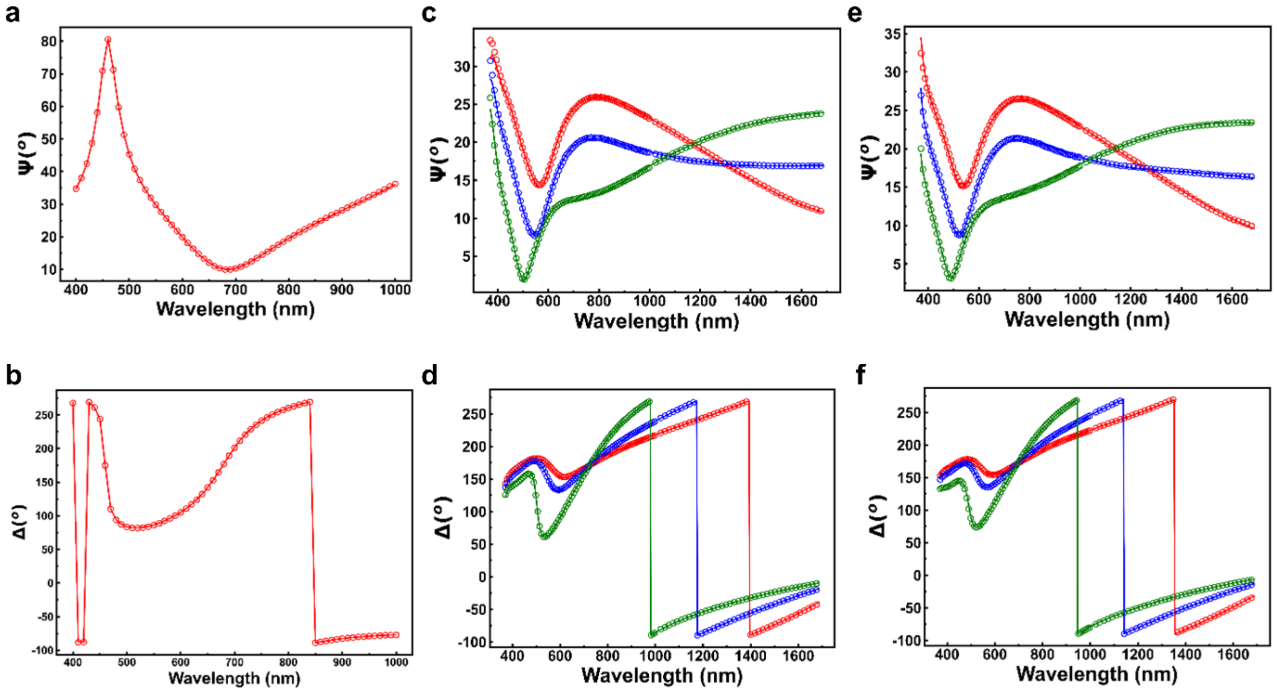

Supplementary Fig. 28. Ellipsometry measurements of 2H-$MoTe_2$, 1T'-$MoTe_2$, and the substrate. (**a, c, e**) Measured (circles) and fitted (lines) ψ for the SiO_2/Si substrate, 2H-$MoTe_2$, and 1T'-$MoTe_2$ at incidence angles 65° (red), 70° (blue), and 75° (green), respectively, (**b, d, f**) Measured (circles) and fitted (lines) Δ for the SiO_2/Si substrate, 2H-$MoTe_2$, and 1T'-$MoTe_2$ at incidence angles 65° (red), 70° (blue), and 75° (green), respectively.

Supplementary Table 1. Comparison of the CVD techniques for the growth of 2H-$MoTe_2$ thin films with dimensions larger than ~0.5 × 0.5 cm^2. Except for this study and Refs.[4,6], the thin films were grown using powder-based horizontal CVD[2,3,5,7,8,73]. The production rate in the unit of [mm/h] is determined based on the lateral dimensions of the wafer [mm] and the time required [h], which should be distinguished from a "growth rate" of a single-crystalline domain[73]. The on-state total sheet conductance ($G_{on,tot} = (I_{on}/V_{ds})(L/W)$ in the unit of [μS]) of 2H-$MoTe_2$ is calculated when the transistor exhibits its maximum current. By comparing $G_{on,tot}$ values, the potential underestimation of I_{on} values owing to variations in channel dimensions can be minimized, enabling fair comparison. The reported FETs[2-8,73] utilizes bottom gate of SiO_2 (except Ref.[2] which employs HfO_2 bottom gate); therefore, the effects of capacitance or $MoTe_2$/dielectric interface on the $G_{on,tot}$ or I_{on}/I_{off} ratio values are similar. We also note that short channel effects on FETs in Refs[2-8,73] are minimal because their channel lengths are larger than ~2 μm.

**Growth aspects**		**Electrical properties**			**Ref.**
**Production rate (mm/h)**	**Possible impurities or residues**	**Contact metal**	**$G_{on,tot}$ (μS)**	**I_{on}/I_{off} ratio**	
**50.5**	**No**	**vdW Au/1T'**	**15.6**	**> 10^5**	**This study**
10	No	1T' edge contact	9.75	~10^3	2
7	No	1T' edge contact	8	~10^4	3
61.5	1T' residues in Raman spectrum	Pd	4.5×10^{-4}	~10^4	4
6.5	Oxide peaks in XPS	Ti/Au	1.3×10^{-2}	~10^2	5
0.35	Required to remove Al_2O_3 layer	1T' edge contact	5	~10^4	6
18	Oxide peaks in XPS	Ti/Au	0.91	~10^3	7
5	No	No transfer curve measurements			8
0.49 at T = 620 °C	Oxide peaks in XPS	1T' edge contact	6.9	~10^4	73

Supplementary Table 2. Comparison of the band structure of $MoTe_2$ polymorphs with those from previous studies.

Phase	Preparation	Measurement	WF (eV)	E_F - E_{VBM}	Ref.
**1T'**	**Te-confined growth**	**UPS**	**4.51**	**0**	**This study**
**2H**	**Te-confined growth**	**UPS**	**4.44**	**0.57**	**This study**
2H	Mechanical exfoliation	UPS	4.42	0.81	[28]
2H	Mechanical exfoliation	UPS	4.35	0.6	[74]
2H	MBE	UPS	4.73	0.83	[75]
2H	MBE	UPS	N/A	0.15	[76]
2H	CVD	UPS	4.85	N/A	[26]
2H	Computational Simulation	DFT calculations	4.29	0.46	[77]
1T'	Computational Simulation	DFT calculations	4.44; 4.46	N/A	[78]

Supplementary Table 3. Benchmarking table for the few-layered 2H-$MoTe_2$-based FETs. To illustrate the reproducibility of the data points, we demonstrated the average values of the tested devices in each report. The best result or the data point extracted solely by one-time measurement of each reference is presented in parentheses. In Ref.[30], the as-fabricated devices were gently annealed in UHV to improve their contact properties. This may lead to a different contact interface compared with those in other studies that did not perform heat treatment following device fabrication. The R_c values for 1T'-$MoTe_2$ contacts[3,6,25,27] presented here include those of 3D-metal-pads/1T'-$MoTe_2$ and 1T'/2H-$MoTe_2$ interfaces. The bottom gate SiO_2 is commonly used in previous studies for 2H-$MoTe_2$ FETs[2,3,5,6,24-30,33,64,79,80], with the exception of cases involving a high-*k* bottom gate[2,26] or BN top gate[64].

**Prep. method for 2H-$MoTe_2$**	**Bottom gate dielectric**	**Contact metal**	**R_c extraction method**	**R_c (kΩ·μm)**	**R_{sh} (kΩ /sq)**	**SBH (meV)**	**I_{on}/I_{off} ratio at RT**	**μ_h at RT ($cm^2V^{-1}s^{-1}$)**	**I_{on} at V_{ds} = -1 V (nA/μm)**	**Ref.**
**CVD**	**300 nm SiO_2**	**1T'**	**TLM**	**1.2 ± 0.5**	**44.3 ± 2.3**	**27.4 (14)**	**1.3 × 10^5 (2.9 × 10^5)**	**21.0 ± 3.3 (29.5)**	**7,820 ± 1,405 (8,814)**	**This study**
CVD	90 nm SiO_2	Ti/Au	N/A	N/A	N/A	N/A	(10^3)	1	(40 at V_d = 0.5 V)	7
	300 nm SiO_2	Ti/Au	TLM	15,600 ± 580	4,670 ± 700	132	10-20	0.6-0.8	5.72 at V_d = -0.1 V	24
		1T' (edge)	N/A	N/A	N/A	30 ± 10	20	7-8	39.6 at V_d = -0.1 V	
	300 nm SiO_2	1T' (vertical; directly synthesized)	TLM	36.4	(125)	N/A	(10^3)	15 (25)	~1,000 at V_d = 0.5 V	25
	35 nm Al_2O_3	Cr/Au	N/A	N/A	N/A	N/A	(10^3)	(0.44)	1.11	26
	12 nm HfO_2	Pd	N/A	N/A	N/A	N/A	(10^3)	2	N/A	2
	285 nm SiO_2	1T' (edge)	N/A	N/A	N/A	250 (DFT)	N/A	N/A	N/A	5
		Ti/Au	TLM	326,500	35,200	N/A	(126)	(0.5)	N/A	
	300 nm SiO_2	Au	Four-point probe	(409)	N/A	150	(10^5)	(4.0)	N/A	27
		1T' (edge)	TLM (irregular line shape)	14	260	22	10^5	16.2	N/A	
	300 nm SiO_2	Pd/Au	N/A	N/A	N/A	N/A	(10^3)	(2)	N/A	3
		1T' (edge)	TLM	1.7	100	N/A	(10^4)	20-24	N/A	
	SiO_2	1T' (edge)	TLM	1.6	N/A	65	1.8 × 10^4	45 ± 2	94 at V_d = 0.1 V	6

MOCVD	300 nm SiO_2	Pd	N/A	N/A	N/A	N/A	(4.8× 10^4)	(0.029)	N/A	4
	SiO_2	Mo_6Te_6 (directly synthesized)	TLM	28,500	53	8.7	(10^3)	(1,139)	N/A	61
	300 nm SiO_2	Te (directly synthesized)	TLM	12.3	0.5	N/A	(6 × 10^2)	(544)	N/A	62
Mechanical exfoliation	285 nm SiO_2	Cr	Four-point probe	(10,000)	N/A	(230)	(10^3)	N/A	N/A	63
	300 nm SiO_2	Ag/Au	N/A	N/A	N/A	N/A	(10^2)	(1.13)	(53)	28
	285 nm SiO_2	Ti/Au	N/A	N/A	N/A	N/A	(2 × 10^3)	(0.3)	N/A	29
	285 nm SiO_2	Ti/Au	N/A	N/A	N/A	N/A	10^2	0.063	N/A	79
	270 nm SiO_2	Ti/Au (Annealed under UHV)	N/A	N/A	N/A	N/A	10^5	20	(7 at V_d = 0.01 V)	30
	100 nm SiO_2	Pd/Ti/Au	N/A	N/A	N/A	(100)	(10^3)	N/A	(1,000 at V_d = -3 V)	31
	Top gate of 10-20 nm BN	Pt (hBN-capped)	Four-point probe	1,000	N/A	N/A	(10^4)	(18)	(285)	64
	SiO_2	Ti	N/A	N/A	N/A	42	(10 at T = 40 K)	N/A	N/A	32
		Cr	N/A	N/A	N/A	41	(10 at T = 40 K)	N/A	N/A	
		Au	N/A	N/A	N/A	30	(10 at T = 40 K)	N/A	N/A	
		Pd	N/A	N/A	N/A	10	(10 at T = 40 K)	N/A	N/A	
	300 nm SiO_2	Pd/Au	N/A	N/A	N/A	(90)	(10^5)	(7.0)	N/A	80
		Cr/Au	N/A	N/A	N/A	300	(10^3)	(1.2)	N/A	
	300 nm SiO_2	Cr	N/A	N/A	N/A	100	2.3 × 10^4 (2.5 × 10^4)	1.0 (1.5)	N/A	33
		Au	N/A	N/A	N/A	75	1.5 × 10^4 (5.7 × 10^4)	0.5 (1.2)	N/A	

Supplementary Table 4. Electrical characteristics of *p*-type WSe_2 FETs based on CVD-grown or mechanically exfoliated flakes and their comparison with the 2H-$MoTe_2$ transistor in this study.

**Prep. method**	**Contact or doping technique**	**Number of layers**	***L* (μm)**	**R_c extraction method**	**R_c (kΩ·μm)**	**n_{2D} (10^{12} cm^{-2})**	**I_{on}/I_{off} ratio**	**I_{on} (μA·$μm^{-1}$)**	**Ref.**
CVD	**vdW Au/1T' contact**	**5-6**	**2**	**TLM**	**1.2**	**7**	**2.9×10^5**	**7.8**	**This study**
	Au contact	1	100	N/A	N/A	2.3	10^6	0.6	[36]
	Synthetic lateral graphene contact	1	10	Y-function	~10^5	N/A	10^7	3	[37]
		1	3	N/A	16.3	N/A	10^6	3.3	[38]
	Nb substitutional doping	1	2	TLM	790	1.4	10^5	0.3	[34]
	V substitutional doping	1	4	N/A	N/A	N/A	10^8	0.5	[35]
	Ni/Au (MOCVD-grown WSe_2)	1	2	N/A	N/A	N/A	10^3–10^4	0.02	[39]
	Synthetic vertical VSe_2 contact	2	0.020	Estimation using R_{on}	0.25-0.54	N/A	1.4×10^2	1580	[50]
	Clean vdW Pt contact	1	1.5	TLM	229	N/A	10^5	7.6	[23]
Mechanical exfoliation	Clean vdW In/Pd alloy	Multilayer	1	TLM	20	1.3	10^5	4	[40]
	Clean vdW Pt contact	Multilayer	4	TLM	3.3	4.8	10^7	25.6	[23]
	Transferred Pt	2	1	Four-point	3.5	N/A	10^6	5	[43]
	Transferred Pt	5	5	N/A	N/A	N/A	10^6	13	[44]

	Transferred Au	Multilayer	2	N/A	N/A	N/A	10^4	2	[45]
	Transferred $NbSe_2$	Multilayer	4.5	N/A	N/A	N/A	10^4	1.7	[46]
	Pt/Au and molecular doping	6-7	0.5	N/A	N/A	N/A	10^6	0.2	[47]
	Pd contact and annealing	7-10	N/A	N/A	N/A	N/A	10^6	1	[48]
	Pd edge contact	3	0.2	N/A	N/A	N/A	10^6	2.3	[81]
	Pt bottom contact and O_3 doping	7	5	N/A	N/A	N/A	10^6	3.5	[41]
	MoO_3 doping	9	5	N/A	N/A	N/A	10^3	1.5	[42]

Supplementary References

1 Yu, Y. *et al.* Phase-Controlled Growth of One-Dimensional Mo_6Te_6 Nanowires and Two-Dimensional MoTe2 Ultrathin Films Heterostructures. *Nano Lett.* **18**, 675-681 (2018).

2 Zhang, Q. *et al.* Simultaneous synthesis and integration of two-dimensional electronic components. *Nat. Electron.* **2**, 164-170 (2019).

3 Xu, X. *et al.* Scaling-up Atomically Thin Coplanar Semiconductor–Metal Circuitry via Phase Engineered Chemical Assembly. *Nano Lett.* **19**, 6845-6852 (2019).

4 Kim, T. *et al.* Wafer-Scale Epitaxial 1T′, 1T′–2H Mixed, and 2H Phases $MoTe_2$ Thin Films Grown by Metal–Organic Chemical Vapor Deposition. *Adv. Mater. Interfaces* **5**, 1800439 (2018).

5 Zhang, X. *et al.* Low Contact Barrier in 2H/1T′ MoTe2 In-Plane Heterostructure Synthesized by Chemical Vapor Deposition. *ACS Appl. Mater. Interfaces* **11**, 12777-12785 (2019).

6 Xu, X. *et al.* Seeded 2D epitaxy of large-area single-crystal films of the van der Waals semiconductor 2H $MoTe_2$. *Science* **372**, 195-200 (2021).

7 Zhou, L. *et al.* Large-Area Synthesis of High-Quality Uniform Few-Layer $MoTe_2$. *J. Am. Chem. Soc.* **137**, 11892-11895 (2015).

8 Yang, L. *et al.* Tellurization Velocity-Dependent Metallic–Semiconducting–Metallic Phase Evolution in Chemical Vapor Deposition Growth of Large-Area, Few-Layer $MoTe_2$. *ACS Nano* **11**, 1964-1972 (2017).

9 Song, Q. J. *et al.* Physical origin of Davydov splitting and resonant Raman spectroscopy of Davydov components in multilayer $MoTe_2$. *Phys. Rev. B* **93**, 115409 (2016).

10 Grzeszczyk, M. *et al.* Raman scattering of few-layers $MoTe_2$. *2D Mater.* **3**, 025010 (2016).

11 Li, Y., Singh, A., Krylyuk, S., Davydov, A. & Jaramillo, R. Near-infrared photonic phase-change properties of transition metal ditellurides. *Proc SPIE Int. Soc. Opt. Eng.* 11085 (2019).

12 Ruppert, C., Aslan, O. B. & Heinz, T. F. Optical Properties and Band Gap of Single- and Few-Layer $MoTe_2$ Crystals. *Nano Lett.* **14**, 6231-6236 (2014).

13 Wilson, J. A. & Yoffe, A. The transition metal dichalcogenides discussion and interpretation of the observed optical, electrical and structural properties. *Adv. Phys.* **18**, 193-335 (1969).

14 Kim, J.-H. *et al.* Carrier multiplication in van der Waals layered transition metal dichalcogenides. *Nat. Commun.* **10**, 5488 (2019).

15 Tauc, J., Grigorovici, R. & Vancu, A. Optical properties and electronic structure of amorphous germanium. *Phys. Status Solidi B* **15**, 627-637 (1966).

16 Munkhbat, B., Wróbel, P., Antosiewicz, T. J. & Shegai, T. O. Optical Constants of Several Multilayer Transition Metal Dichalcogenides Measured by Spectroscopic Ellipsometry in the 300–1700 nm Range: High Index, Anisotropy, and Hyperbolicity. *ACS Photonics* **9**, 2398-2407 (2022).

17 Green, M. A. Self-consistent optical parameters of intrinsic silicon at 300K including temperature coefficients. *Sol. Energy Mater. Sol. Cells* **92**, 1305-1310 (2008).

18 Amotchkina, T., Trubetskov, M., Hahner, D. & Pervak, V. Characterization of e-beam evaporated Ge, YbF_3, ZnS, and LaF_3 thin films for laser-oriented coatings. *Appl. Opt.* **59**, A40-A47 (2020).

19 Ruppert, C., Aslan, B. & Heinz, T. F. Optical Properties and Band Gap of Single- and Few-Layer $MoTe_2$ Crystals. *Nano Lett.* **14**, 6231-6236 (2014).

20 Liu, M., Liu, W. & Wei, Z. $MoTe_2$ Saturable Absorber With High Modulation Depth for Erbium-Doped Fiber Laser. *J. Light. Technol.* **37**, 3100-3105 (2019).

21 Cao, H. *et al.* Efficient and Fast All-Optical Modulator with In Situ Grown $MoTe_2$ Nanosheets on Silicon. *ACS Appl. Nano Mater.* **6**, 838-845 (2023).

22 Bie, Y.-Q. *et al.* A $MoTe_2$-based light-emitting diode and photodetector for silicon photonic integrated circuits. *Nat. Nanotechnol.* **12**, 1124-1129 (2017).

23 Wang, Y. *et al.* P-type electrical contacts for 2D transition-metal dichalcogenides. *Nature* **610**, 61-66 (2022).

24 Ma, R. *et al.* $MoTe_2$ Lateral Homojunction Field-Effect Transistors Fabricated using Flux-Controlled Phase Engineering. *ACS Nano* **13**, 8035-8046 (2019).

25 Yang, S. *et al.* Large-Scale Vertical 1T′/2H $MoTe_2$ Nanosheet-Based Heterostructures for Low Contact Resistance Transistors. *ACS Appl. Nano Mater.* **3**, 10411-10417 (2020).

26 Park, Y. J., Katiyar, A. K., Hoang, A. T. & Ahn, J.-H. Controllable P- and N-Type Conversion of $MoTe_2$ via Oxide Interfacial Layer for Logic Circuits. *Small* **15**, 1901772 (2019).

27 Sung, J. H. *et al.* Coplanar semiconductor–metal circuitry defined on few-layer $MoTe_2$ via polymorphic heteroepitaxy. *Nature Nanotechnol.* **12**, 1064 (2017).

28 Zheng, X. *et al.* Enormous enhancement in electrical performance of few-layered $MoTe_2$ due to Schottky barrier reduction induced by ultraviolet ozone treatment. *Nano Res.* **13**, 952-958 (2020).

29 Lin, Y.-F. *et al.* Ambipolar $MoTe_2$ Transistors and Their Applications in Logic Circuits. *Adv. Mater.* **26**, 3263-3269 (2014).

30 Pradhan, N. R. *et al.* Field-effect transistors based on few-layered α-$MoTe_2$. *ACS Nano* **8**, 5911-5920 (2014).

31 Haratipour, N. & Koester, S. Multi-layer $MoTe_2$ p-channel MOSFETs with high drive current. *72nd Dev. Res. Conf.* 171-172 (2014).

32 Townsend, N. J., Amit, I., Craciun, M. F. & Russo, S. Sub 20 meV Schottky barriers in metal/MoTe 2 junctions. *2D Mater.* **5**, 025023 (2018).

33 Yin, L. *et al.* Ultrahigh sensitive $MoTe_2$ phototransistors driven by carrier tunneling. *Appl. Phys. Lett.* **108**, 043503 (2016).

34 Vu, V. T. *et al.* One-Step Synthesis of $NbSe_2$/Nb-Doped-WSe_2 Metal/Doped-Semiconductor van der Waals Heterostructures for Doping Controlled Ohmic Contact. *ACS Nano* **15**, 13031-13040 (2021).

35 Li, S. *et al.* Tunable Doping of Rhenium and Vanadium into Transition Metal Dichalcogenides for Two-Dimensional Electronics. *Adv. Sci.* **8**, 2004438 (2021).

36 Gao, Y. *et al.* Ultrafast Growth of High-Quality Monolayer WSe_2 on Au. *Adv. Mater.* **29**, 1700990 (2017).

37 Tang, H.-L. *et al.* Multilayer Graphene–WSe_2 Heterostructures for WSe_2 Transistors. *ACS Nano* **11**, 12817-12823 (2017).

38 Chu, C.-H. *et al.* End-Bonded Metal Contacts on WSe_2 Field-Effect Transistors. *ACS Nano* **13**, 8146-8154 (2019).

39 Kozhakhmetov, A. *et al.* Scalable BEOL compatible 2D tungsten diselenide. *2D Mater.* **7**, 015029 (2020).

40 Wang, Y. *et al.* Van der Waals contacts between three-dimensional metals and two-dimensional semiconductors. *Nature* **568**, 70-74 (2019).

41 Yang, S., Lee, G. & Kim, J. Selective p-Doping of 2D WSe_2 via UV/Ozone Treatments and Its Application in Field-Effect Transistors. *ACS Appl. Mater. Interfaces* **13**, 955-961 (2021).

42 Zhou, C. *et al.* Carrier Type Control of WSe_2 Field-Effect Transistors by Thickness Modulation and MoO3 Layer Doping. *Adv. Funct. Mater.* **26**, 4223-4230 (2016).

43 Jung, Y. *et al.* Transferred via contacts as a platform for ideal two-dimensional transistors. *Nat. Electron.* **2**, 187-194 (2019).

44 Wang, J. *et al.* Steep Slope p-type 2D WSe_2 Field-Effect Transistors with Van Der Waals Contact and Negative Capacitance. *2018 IEEE International Electron Devices Meeting (IEDM).* pp. 22.3.1-22.3.4 (2018).

45 Kong, L. *et al.* Doping-free complementary WSe_2 circuit via van der Waals metal integration. *Nat. Commun.* **11**, 1866 (2020).

46 Sata, Y. *et al.* N- and p-type carrier injections into WSe_2 with van der Waals contacts of two-dimensional materials. *Jpn. J. Appl. Phys.* **56**, 04CK09 (2017).

47 Si, M. *et al.* Steep-Slope WSe_2 Negative Capacitance Field-Effect Transistor. *Nano Lett.* **18**, 3682-3687 (2018).

48 Smyth, C. M. *et al.* Engineering the Palladium–WSe_2 Interface Chemistry for Field Effect Transistors with High-Performance Hole Contacts. *ACS Appl. Nano Mater.* **2**, 75-88 (2019).

49 Shen, P.-C. *et al.* Ultralow contact resistance between semimetal and monolayer semiconductors. *Nature* **593**, 211-217 (2021).

50 Wu, R. *et al.* Bilayer tungsten diselenide transistors with on-state currents exceeding 1.5 milliamperes per micrometre. *Nat. Electron.* **5**, 497-504 (2022).

51 Kim, H.-g. & Choi, H. J. Thickness dependence of work function, ionization energy, and electron affinity of Mo and W dichalcogenides from DFT and GW calculations. *Phys. Rev. B* **103**, 085404 (2021).

52 Liu, Y., Stradins, P. & Wei, S.-H. Van der Waals metal-semiconductor junction: Weak Fermi level pinning enables effective tuning of Schottky barrier. *Sci. Adv.* **2**, e1600069 (2016).

53 Zheng, X. *et al.* Controlled Layer-by-Layer Oxidation of $MoTe_2$ via O_3 Exposure. *ACS Appl. Mater. Interfaces* **10**, 30045-30050 (2018).

54 Zhang, K. *et al.* Epitaxial substitution of metal iodides for low-temperature growth of two-dimensional metal chalcogenides. *Nat. Nanotechnol.* **18**, 448-455 (2023).

55 Kwon, G. *et al.* Interaction- and defect-free van der Waals contacts between metals and two-dimensional semiconductors. *Nat. Electron.* **5**, 241-247 (2022).

56 Kong, L. *et al.* Wafer-scale and universal van der Waals metal semiconductor contact. *Nat. Commun.* **14**, 1014 (2023).

57 Cui, J. *et al.* Transport evidence of asymmetric spin–orbit coupling in few-layer superconducting $1T_d$-$MoTe_2$. *Nat. Commun.* **10**, 2044 (2019).

58 Wang, Q. *et al.* Observation of Weak Anti-Localization and Electron-Electron Interaction on Few-Layer 1T′-$MoTe_2$ Thin Films. *Chin. Phys. Lett.* **35**, 077303 (2018).

59 Kuiri, M., Das, S., Muthu, D., Das, A. & Sood, A. Thickness dependent transition from the 1T′ to Weyl semimetal phase in ultrathin $MoTe_2$: electrical transport, noise and Raman studies. *Nanoscale* **12**, 8371-8378 (2020).

60 Song, S. *et al.* Wafer-scale production of patterned transition metal ditelluride layers for two-dimensional metal–semiconductor contacts at the Schottky–Mott limit. *Nat. Electron.* **3**, 207-215 (2020).

61 Lee, R. S. *et al.* van der Waals Epitaxy of High-Mobility Polymorphic Structure of Mo_6Te_6 Nanoplates/$MoTe_2$ Atomic Layers with Low Schottky Barrier Height. *ACS Nano* **13**, 642-648 (2019).

62 Choi, D. *et al.* Directly grown Te nanowire electrodes and soft plasma etching for high-performance $MoTe_2$ field-effect transistors. *Appl. Surf. Sci.* **565**, 150521 (2021).

63 Kim, C. *et al.* Fermi Level Pinning at Electrical Metal Contacts of Monolayer Molybdenum Dichalcogenides. *ACS Nano* **11**, 1588-1596 (2017).

64 Larentis, S. *et al.* Reconfigurable Complementary Monolayer $MoTe_2$ Field-Effect Transistors for Integrated Circuits. *ACS Nano* **11**, 4832-4839 (2017).

65 Schranghamer, T. F. *et al.* Ultrascaled Contacts to Monolayer MoS_2 Field Effect Transistors. *Nano Lett.* **23**, 3426-3434 (2023).

66 English, C. D., Shine, G., Dorgan, V. E., Saraswat, K. C. & Pop, E. Improved Contacts to MoS_2 Transistors by Ultra-High Vacuum Metal Deposition. *Nano Lett.* **16**, 3824-3830 (2016).

67 Mleczko, M. J. *et al.* Contact Engineering High-Performance n-Type $MoTe_2$ Transistors. *Nano Lett.* **19**, 6352-6362 (2019).

68 Kaasbjerg, K., Thygesen, K. S. & Jacobsen, K. W. Phonon-limited mobility in n-type single-layer MoS_2 from first principles. *Phys. Rev. B* **85**, 115317 (2012).

69 Jariwala, D. *et al.* Band-like transport in high mobility unencapsulated single-layer MoS_2 transistors. *Appl. Phys. Lett.* **102**, 173107 (2013).

70 Liu, H., Neal, A. T. & Ye, P. D. Channel Length Scaling of MoS_2 MOSFETs. *ACS Nano* **6**, 8563-8569 (2012).

71 Ovchinnikov, D., Allain, A., Huang, Y.-S., Dumcenco, D. & Kis, A. Electrical Transport Properties of Single-Layer WS_2. *ACS Nano* **8**, 8174-8181 (2014).

72 Tosun, M. *et al.* Air-Stable n-Doping of WSe_2 by Anion Vacancy Formation with Mild Plasma Treatment. *ACS Nano* **10**, 6853-6860 (2016).

73 Xu, X. *et al.* Millimeter-Scale Single-Crystalline Semiconducting $MoTe_2$ via Solid-to-Solid Phase Transformation. *J. Am. Chem. Soc.* **141**, 2128-2134 (2019).

74 Cho, Y. *et al.* Fully Transparent p-$MoTe_2$ 2D Transistors Using Ultrathin MoO_x/Pt Contact Media for Indium-Tin-Oxide Source/Drain. *Adv. Funct. Mater.* **28**, 1801204 (2018).

75 Diaz, H. C., Ma, Y., Chaghi, R. & Batzill, M. High density of (pseudo) periodic twin-grain boundaries in molecular beam epitaxy-grown van der Waals heterostructure: $MoTe_2/MoS_2$. *Appl. Phys. Lett.* **108**, 191606 (2016).

76 He, Q. *et al.* Molecular Beam Epitaxy Scalable Growth of Wafer-Scale Continuous Semiconducting Monolayer $MoTe_2$ on Inert Amorphous Dielectrics. *Adv. Mater.* **31**, 1901578 (2019).

77 Zhang, C. *et al.* Systematic study of electronic structure and band alignment of monolayer transition metal dichalcogenides in Van der Waals heterostructures. *2D Mater.* **4**, 015026 (2016).

78 Sun, Y. *et al.* Interface-mediated noble metal deposition on transition metal dichalcogenide nanostructures. *Nat. Chem.* **12**, 284-293 (2020).

79 Chang, Y.-M. *et al.* Reversible and Precisely Controllable p/n-Type Doping of $MoTe_2$ Transistors through Electrothermal Doping. *Adv. Mater.* **30**, 1706995 (2018).

80 Aftab, S. *et al.* Formation of an $MoTe_2$ based Schottky junction employing ultra-low and high resistive metal contacts. *RSC Adv.* **9**, 10017-10023 (2019).

81 Abuzaid, H., Cheng, Z., Li, G., Cao, L. & Franklin, A. D. Unanticipated Polarity Shift in Edge-Contacted Tungsten-Based 2D Transition Metal Dichalcogenide Transistors. *IEEE Electron Device Lett.* **42**, 1563-1566 (2021).
